# Proliferation of hydrocarbon-degrading microbes at the bottom of the Mariana Trench

**DOI:** 10.1186/s40168-019-0652-3

**Published:** 2019-04-12

**Authors:** Jiwen Liu, Yanfen Zheng, Heyu Lin, Xuchen Wang, Meng Li, Yang Liu, Meng Yu, Meixun Zhao, Nikolai Pedentchouk, David J. Lea-Smith, Jonathan D. Todd, Clayton R. Magill, Wei-Jia Zhang, Shun Zhou, Delei Song, Haohui Zhong, Yu Xin, Min Yu, Jiwei Tian, Xiao-Hua Zhang

**Affiliations:** 1 MOE Key Laboratory of Marine Genetics and Breeding, College of Marine Life Sciences, Ocean University of China, Qingdao, 266003 China; 2 Laboratory for Marine Ecology and Environmental Science, Qingdao National Laboratory for Marine Science and Technology, Qingdao, 266237 China; 3 Key Laboratory of Marine Chemistry Theory and Technology, Ministry of Education, Qingdao, 266100 China; 4 Institute for Advanced Study, Shenzhen University, Shenzhen, 518060 China; 5 School of Environmental Sciences, University of East Anglia, Norwich, NR4 7TJ UK; 6 School of Biological Sciences, University of East Anglia, Norwich, NR4 7TJ UK; 7 Lyell Centre, Heriot-Watt University, Edinburgh, EH14 4AS UK; 8 Laboratory of Deep Sea Microbial Cell Biology, Institute of Deep Sea Science and Engineering, Chinese Academy of Sciences, Sanya, 572000 China; 9 Key Laboratory of Physical Oceanography, Ministry of Education, Ocean University of China, Qingdao, 266100 China; 10 Marine Dynamic Process and Climate Function Laboratory, Qingdao National Laboratory for Marine Science and Technology, Qingdao, 266237 China

**Keywords:** Challenger Deep, Mariana Trench, Hadal water, Metagenomics, Microbial community, Hydrocarbon degradation, Hydrocarbon biosynthesis

## Abstract

**Background:**

The Mariana Trench is the deepest known site in the Earth’s oceans, reaching a depth of ~ 11,000 m at the Challenger Deep. Recent studies reveal that hadal waters harbor distinctive microbial planktonic communities. However, the genetic potential of microbial communities within the hadal zone is poorly understood.

**Results:**

Here, implementing both culture-dependent and culture-independent methods, we perform extensive analysis of microbial populations and their genetic potential at different depths in the Mariana Trench. Unexpectedly, we observed an abrupt increase in the abundance of hydrocarbon-degrading bacteria at depths > 10,400 m in the Challenger Deep. Indeed, the proportion of hydrocarbon-degrading bacteria at > 10,400 m is the highest observed in any natural environment on Earth. These bacteria were mainly *Oleibacter*, *Thalassolituus*, and *Alcanivorax* genera, all of which include species known to consume aliphatic hydrocarbons. This community shift towards hydrocarbon degraders was accompanied by increased abundance and transcription of genes involved in alkane degradation. Correspondingly, three *Alcanivorax* species that were isolated from 10,400 m water supplemented with hexadecane were able to efficiently degrade *n*-alkanes under conditions simulating the deep sea, as did a reference *Oleibacter* strain cultured at atmospheric pressure. Abundant *n-*alkanes were observed in sinking particles at 2000, 4000, and 6000 m (averaged 23.5 μg/gdw) and hadal surface sediments at depths of 10,908, 10,909, and 10,911 m (averaged 2.3 μg/gdw). The δ^2^H values of *n-*C_16/18_ alkanes that dominated surface sediments at near 11,000-m depths ranged from − 79 to − 93‰, suggesting that these sedimentary alkanes may have been derived from an unknown heterotrophic source.

**Conclusions:**

These results reveal that hydrocarbon-degrading microorganisms are present in great abundance in the deepest seawater on Earth and shed a new light on potential biological processes in this extreme environment.

**Electronic supplementary material:**

The online version of this article (10.1186/s40168-019-0652-3) contains supplementary material, which is available to authorized users.

## Background

The hadal zone (6000–11,000 m) is the deepest part of the marine environment and is composed almost exclusively of trenches. Hadal zone planktonic microbial communities have recently been investigated in the Mariana [1–3], Puerto Rico [4], Japan [5], and Kermadec Trenches [6] due to rapid technological progress in deep-sea sampling. The hadal and abyssal (4000–6000 m) waters differ significantly in their micro- and macro-community compositions [1, 5, 7]. Specifically, heterotrophic *Gammaproteobacteria* and *Bacteroidetes* are dominant in the hadal ocean, compared to *Thaumarchaeota* in the abyssal ocean [1, 5, 6]. There are only a few depth profile studies of microbial communities in the hadal zone, and thus, little is known of the variability in community composition in this unique environment. In the Challenger Deep of the Mariana Trench, Nunoura et al. demonstrated via 16S rRNA gene sequencing that *Gammaproteobacteria* were the dominant class at ~ 10,257 m, comprising 62.7% of the total microbial population, compared to 21.6% and 1.7% at 9000 and 7998 m, respectively [1]. Moreover, *Oceanospirillales* comprised 78.1% of the total *Gammaproteobacteria* population at 10,257 m, unlike at 9000 m where *Pseudomonadales* dominated. Tarn et al. also confirmed the dominance of *Gammaproteobacteria* in both the Challenger (~ 10,918 m) and Sirena (~ 10,667 m) Deeps of the Mariana Trench, using 16S rRNA gene sequencing [2]. The drivers for this major microbial community shift at depths > 10,000 m are undetermined, since studies analyzing the genetic potential of microbes [8] and their abundance at the genus and species level at these depths are limited.

To address these knowledge gaps, we conducted the most detailed analysis to date of microbial populations and functional capacities in the Mariana Trench water samples from different depths by metagenome sequencing, 16S rRNA gene amplicon, and gene probe analyses. An unexpected enrichment for hydrocarbon-degrading bacteria, along with an increase in the abundance and transcription of hydrocarbon-degrading genes, was found at depths near the trench bottom (10,400 m and 10,500 m). Based on this information, we experimentally tested and confirmed the hydrocarbon degradation activity of bacterial strains isolated from 10,400 m water supplemented with hexadecane under conditions simulating the deep ocean. Stable hydrogen isotope analysis of surface sediments at the bottom of the trench suggests that *n-*C_16/18_ alkanes, the dominant hydrocarbons in these samples, are derived from an unknown biological source, and may support hydrocarbon-degrading microbial populations.

## Results and discussion

### Microbial communities vary throughout the Mariana Trench water column

To determine the vertical distribution of microbial populations in the Challenger Deep, shotgun metagenome sequencing and 16S rRNA gene amplicon analyses were performed on free-living (0.22–3 μm) (FL) and particle-associated (> 3 μm) (PA) communities collected from surface waters and from depths of 4000 m, 9600 m, 10,400 m, and 10,500 m (Additional file 1: Table S1 and Figure S1). Also, environmental analysis was performed on samples collected from 4 to 8320 m, which demonstrated that hadal and abyssal waters shared many comparable physiochemical factors, including temperature, salinity, oxygen, and nutrients (Additional file 1: Table S2).

Quantitative PCR assays showed that the 16S rRNA gene copy numbers decreased significantly from 0 (7.73 × 10^7^ copies/L) to 4000 m (1.21 × 10^7^ copies/L), but then increased with depth to 7.43 × 10^7^, 7.58 × 10^7^, and 7.82 × 10^7^ copies/L at 9600, 10,400, and 10,500 m, respectively (Additional file 1: Figure S2). Similarly, Nunoura et al. showed that the abundance of 16S rRNA gene decreased along with depth before 6000 m but then increased slightly from 6000 m to 10,257 m except for 9000 m [1]. In this study, the increase of 16S rRNA gene copy number in trench bottom water compared to 4000 m may be due to increased *Gammaproteobacteria* populations (see below), which harbor ~5.7 copies of 16S rRNA gene on average, far higher than other *Proteobacteria* [9], suggesting that 16S rRNA gene copy number is not always a good proxy of cell number.

**Fig. 1:**
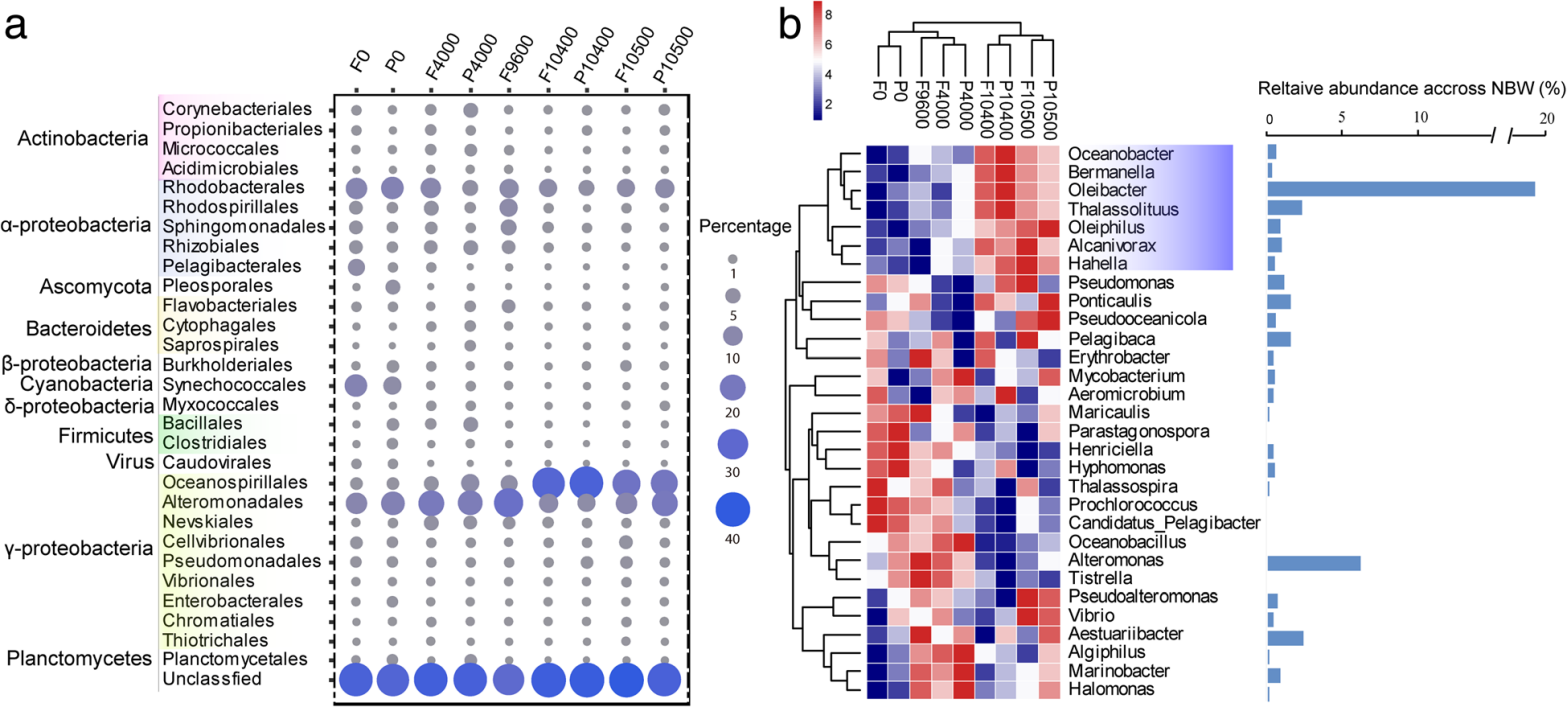
The composition of dominant microbial groups (top 30 abundant across all samples). **a** Microbial community shifts at order level with depth**. b** Microbial community shifts at genus level with depth. The analysis is based on the metagenomic data annotation against the NCBI-nr database. Sample names are defined by size fraction and sampling depth, e.g., FL10500 is the free-living fraction at 10,500 m. Dot size in the panel **a** is proportional to the relative abundance of microbial groups. The relative abundance at the genus level was rank transformed. The genera shadowed in blue indicate significant enrichment in the four NBW samples. The bar plot at the right side shows the average proportion (across the four NBW samples) of each genus in the total microbial population

Taxonomic profiling of metagenomic data based on the NCBI-nr database revealed that bacteria accounted for the majority of the population at all depths in both FL and PA samples, compared to archaea and eukaryotes (Additional file 1: Figure S3a). As expected, *Proteobacteria*, particularly *Alphaproteobacteria* and *Gammaproteobacteria*, were the predominant organisms at all depths, whereas *Cyanobacteria* were relatively abundant only in surface water, accounting for 13.9% and 7.3% of the FL and PA samples, respectively (Additional file 1: Figure S3b and 3c). The relative abundance of *Gammaproteobacteria* increased with depth from 21.3% at 0 m to 31.3% at 4000 m, 38.4% at 9600 m, and 49.2% at both 10,400 m and 10,500 m (Additional file 1: Table S3). Similar to Nunoura et al. [1], the relative abundance of *Oceanospirillales* increased significantly (*P* < 0.01) in the two near bottom water (NBW; ~ 300–400 m from the seafloor) samples, comprising 61.4% of the gammaproteobacterial genes (5.6-fold increase compared to 9600 m) (Fig. 1a). This dominance of *Oceanospirillales* in NBW was confirmed by analyses of 16S rRNA gene recovered from both the metagenomic and amplicon sequencing (Additional file 1: Figure S4), suggesting that a bacterial niche boundary exits at that depth. Several bacterial lineages such as *Pelagibacterales* differed in relative abundance among different methods used (Additional file 1: Figure S4). Such differences may have resulted from biases in PCR amplification, variation in gene copy number, and genome size [10].

Next, we analyzed the genus-level composition to identify the genera that are significantly enriched in NBW samples. According to the metagenomic data annotated against the NCBI-nr database, seven genera were significantly more abundant in NBW than at other depths (*P* < 0.05) (Fig. 1b). All belonged to the order *Oceanospirillales* and included *Oleibacter* (19.5 ± 6.5%), *Thalassolituus* (2.5 ± 0.7%), *Alcanivorax* (1.0 ± 0.4%), *Oleiphilus* (0.9 ± 0.7%), *Oceanobacter* (0.6 ± 0.2%), *Hahella* (0.5 ± 0.4%), and *Bermanella* (0.3 ± 0.1%). 16S rRNA gene amplicon analysis also confirmed the predominance of *Oleibacter* in NBW (37.8 ± 8.2%). This is the first environment where *Oleibacter* has been reported as the most abundant genus.

### *Oceanospirillales* species encode genes involved in alkane degradation

**Fig. 2:**
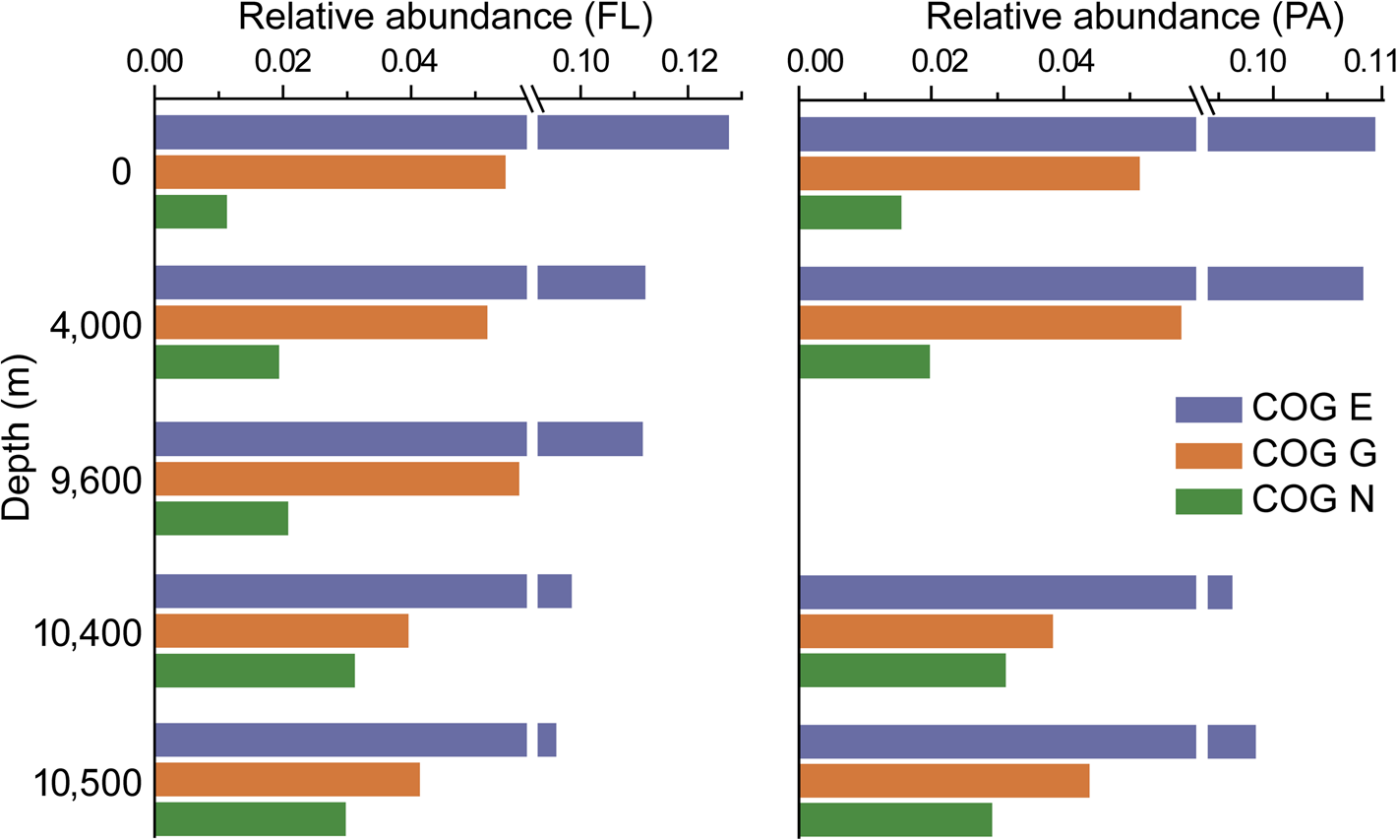
The relative abundance of three major COG categories related to amino acid transport and metabolism (E), carbohydrate transport and metabolism (G), and cell motility (N). FL, free living; PA, particle associated

To explore the genetic potential in *Oceanospirillales* that dominate the NBW environment, we compared the metagenomic data against the COG and KEGG databases. The NBW harbored a significantly higher abundance of genes encoding cell motility proteins compared to other depths (*P* < 0.05) (Fig. 2 and Additional file 1: Figure S5). Predominant among these were genes encoding methyl-accepting chemotaxis proteins (MCP) and type IV pilus assembly genes that function in cell adhesion and twitching motility [11] (Additional file 1: Figure S6), which were mainly attributed to *Oleibacter* (Additional file 1: Figure S7), a genus which includes species known to degrade hydrocarbons [12, 13]. MCPs have been found to play an important role in sensing various substrates including alkanes [14] and thus may facilitate hydrocarbon degradation by *Oleibacter* species. MCP and pilus genes were highly transcribed in microbial populations following the Deepwater Horizon oil spill [15, 16], further supporting a key role for these genes in microbial hydrocarbon degradation. NBW harbored a lower abundance of genes related to carbohydrate and protein metabolism (COG E and G; Fig. 2), as well as genes involved in the cycling of carbon and sulfur (Additional file 1: Figure S8), suggesting the microbial population in NBW might utilize alternative carbon sources.

**Fig. 3:**
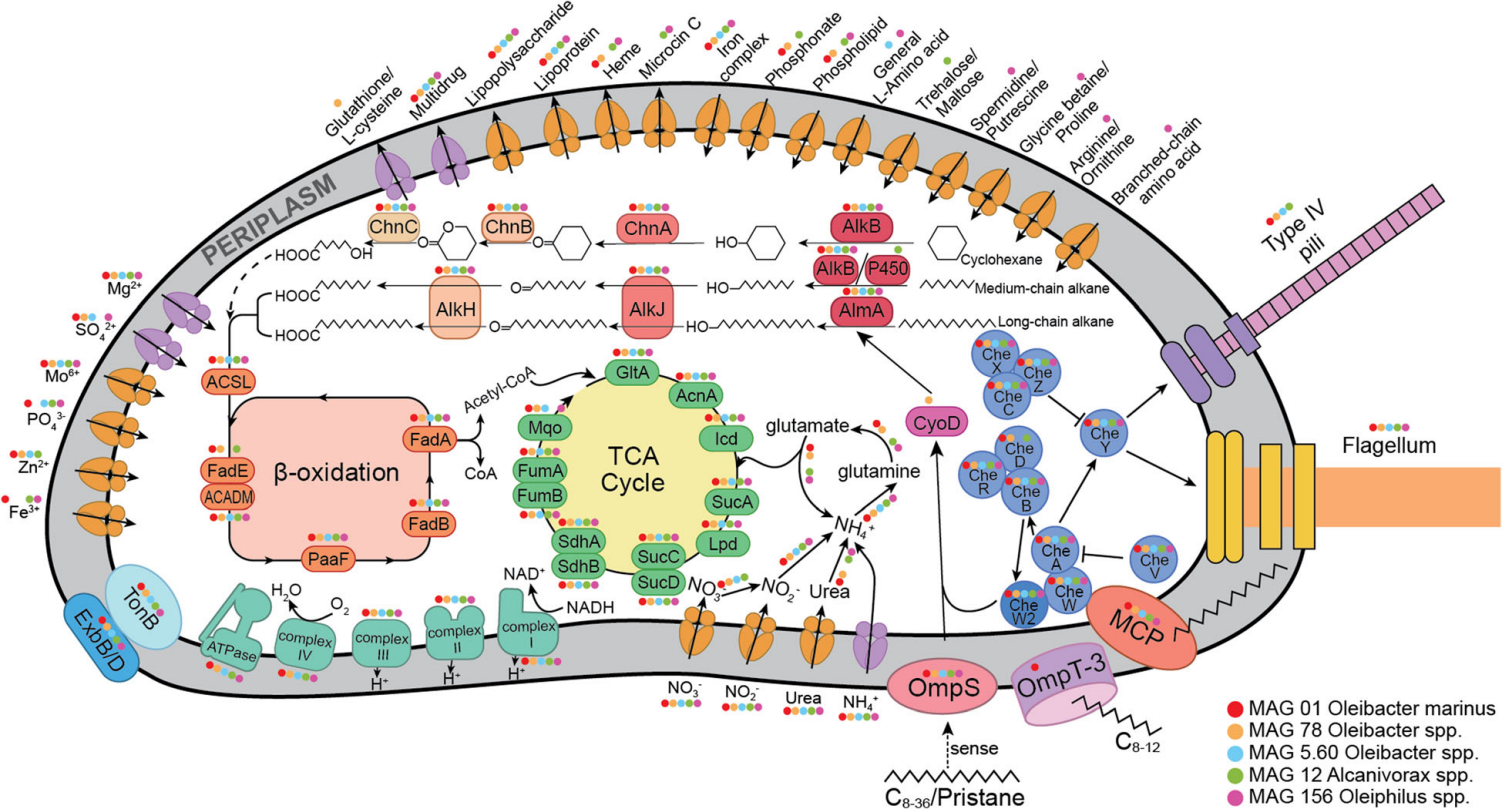
Hydrocarbon degradation pathways are present in the five high-quality metagenome assembled genomes (MAGs) belonging to *Oceanospirillales*. Predicted metabolic pathways and corresponding proteins in a given bacterial genome are indicated by colored dots to demonstrate the comparison between MAGs. The predictions are based on RAST and KEGG annotation. All five MAGs contained potential complete set of enzymes required for alkane degradation

To predict hydrocarbon degradation pathways in the abundant NBW *Oceanospirillales* bacteria described above, we constructed 43 high-quality genomic bins, hereafter referred to as metagenome assembled genomes (MAGs), from the combined contigs of the four NBW samples with a contaminant threshold of 10% and a completeness threshold of 80%. These include 33 *Proteobacteria* and three *Actinobacteria* MAGs; the former contain 19 *Alpha-*, nine *Gamma-*, and five *Deltaproteobacteria* MAGs (Additional file 1: Table S4). Five of the *Gammaproteobacteria* MAGs, termed MAG 01 (completeness 90.8%), 78 (completeness 87.7%), 5.60 (completeness 83.2%), 12 (completeness 93.2%), and 156 (completeness 97.8%), are related to *Oceanospirillales*. The first three MAGs (01, 78, and 5.60) are phylogenetically similar to *Oleibacter*, while the latter two are similar to *Alcanivorax* and *Oleiphilus*, respectively. The three *Oleibacter* MAGs (01, 78, and 5.60) showed an average nucleotide identity of 74.7% to each other, and 98.6%, 77.2%, and 73.6% to *Oleibacter marinus* DSM 24913, respectively. Strain DSM 24913 is a hydrocarbonoclastic bacterium isolated from a crude oil enrichment of Indonesian seawater [12] and the only cultured species available in this genus. Our analyses suggest that MAG 01, 78, and 5.60 represent different *Oleibacter* species, and MAG 01 is affiliated to *O. marinus.* This was further verified by the high 16S rRNA gene sequence similarity (99.0%) between MAG 01 and *O. marinus* DSM 24913. Mapping raw reads to the MAGs showed that MAG 01 was the dominant *Oleibacter* clade in NBW; its relative abundance was the highest among all MAGs and accounted for 11.6 ± 2.4% of all NBW reads (Additional file 1: Figure S9). These five *Oceanospirillales* MAGs possess predicted pathways for both medium-chain alkane 1-monooxygenase (AlkB) and long-chain alkane monooxygenase (AlmA)-mediated alkane degradation, for cyclohexane degradation, MCP, and most other chemotactic genes (Fig. 3). Thus, metagenomic predictions suggest that Challenger Deep NBW *Oceanospirillales* bacteria can metabolize medium-chain, long-chain, and some cyclic hydrocarbons and that such molecules may be important sources of carbon and/or energy in such environments.

To the best of our knowledge, the proportion of *Oceanospirillales-*derived alkane degraders in NBW (~ 20% in the metagenome and ~ 38% in the 16S rRNA gene amplicon; Additional file 1: Figure S4a and c) is higher than in any other natural environments observed on Earth. However, the predominance of *Oceanospirillales*, along with MCP, *alkB*, and type IV pilus assembly genes in NBW, resembles the situation (dominant microbial communities and functional genes) in the Deepwater Horizon spill (DWH) [15–19], suggesting that the microorganisms in these two distinct environments most likely share similar hydrocarbon-degrading pathways. However, the predominant DWH *Oceanospirillales* showed high sequence similarity to *Candidatus* Bermanella macondoprimitus and/or *Candidatus* Oceanospirillales desum [20, 21]. Although *O. marinus* was detected in *n*-hexadecane enrichments using water collected from oil plume at 1000 m after the DWH, it only represented 3% of the total sequences [22].

### Abundance and expression of alkane degradation genes increase in near bottom waters

**Fig. 4:**
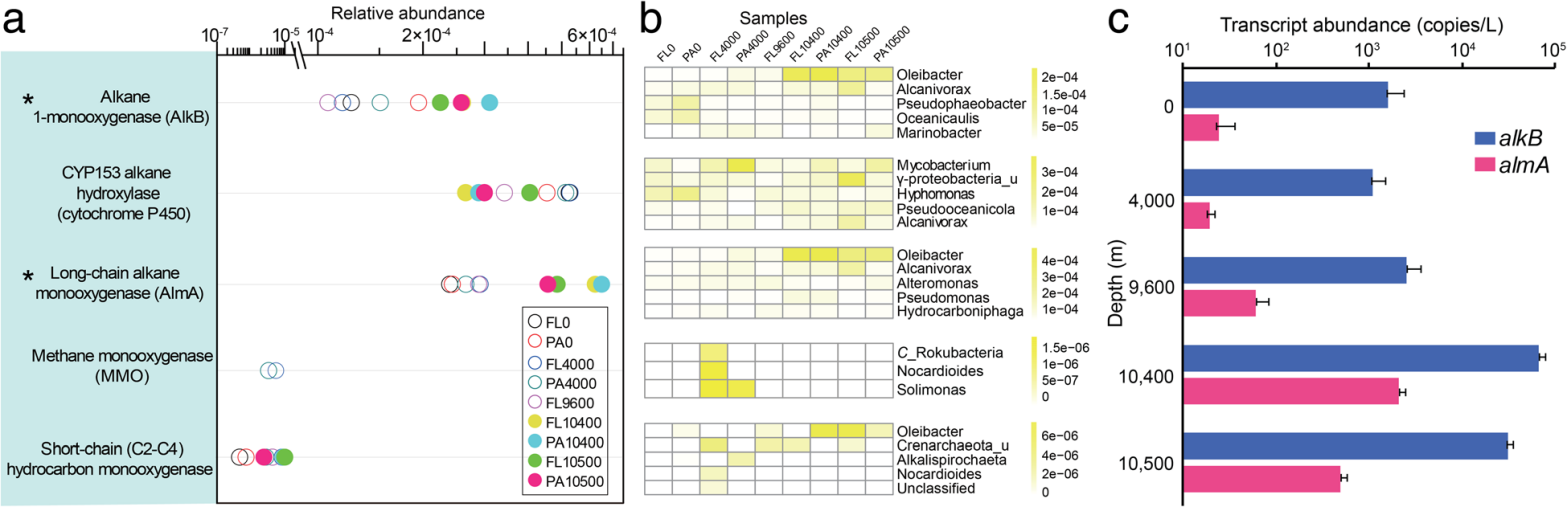
The relative abundance and expression of genes associated with aliphatic hydrocarbon degradation increase in NBW. **a** The relative abundance of genes at different depths. **b** The top five affiliated genera of each gene are shown in the heat map. **c** Transcript abundance of *alkB* and *almA* in free-living samples from different depths. Asterisks in panel **a** indicate statistically significant difference in gene abundance between the two zones

The data above suggest that microbial populations in NBW have the potential to degrade hydrocarbons. To test this, we first investigated the abundance of putative hydrocarbon-degrading genes at different water depths (Fig. 4). Interestingly, the relative abundance of genes encoding AlkB, AlmA (Fig. 4a), and several enzymes for sensing and transporting alkanes (Additional file 1: Figure S10) increased significantly in NBW compared to other water depths. The absolute abundance of *alkB* and *almA* and the proportion of microbial cells possessing them exhibited similar trends (Additional file 1: Figure S11). These numbers displayed positive relationships with the relative abundance of *Oceanospirillales* (*r* > 0.85, *P* < 0.01), notably *Oleibacter* (Fig. 4b and Additional file 1: Table S5). The increase of *alkB* and *almA* in NBW was similar to that of MCP (Additional file 1: Figure S6). The abundance of the other known aliphatic hydrocarbon degradation genes was not statistically different among samples from different depths (Fig. 4a). In addition, many aromatic hydrocarbon degradation genes (e.g., for catechol, protocatechuate and gentisate metabolism) were lower in NBW than in other water depths (Additional file 1: Figure S12). Overall, this suggests that alkanes are the main hydrocarbon source utilized by *Oceanospirillales* species.

To determine whether increases in the transcription of *alkB* and *almA* corelate with their gene abundance, we performed RT-qPCR on 0, 4000, 9600, 10,400, and 10,500 m samples. *AlkB* and *almA* genes were highly transcribed in NBW compared to 0, 4000, and 9600 m (up to 42, 61, 26 and 86, 109, 34-fold increased, respectively) (Fig. 4c), and 81.5–100% of transcripts were from *Oleibacter*. This further supports the hypothesis that *Oceanospirillales* species are degrading both medium- and long-chain alkanes in this environment.

### Near bottom water isolates degrade alkanes under physiologically relevant conditions

**Fig. 5:**
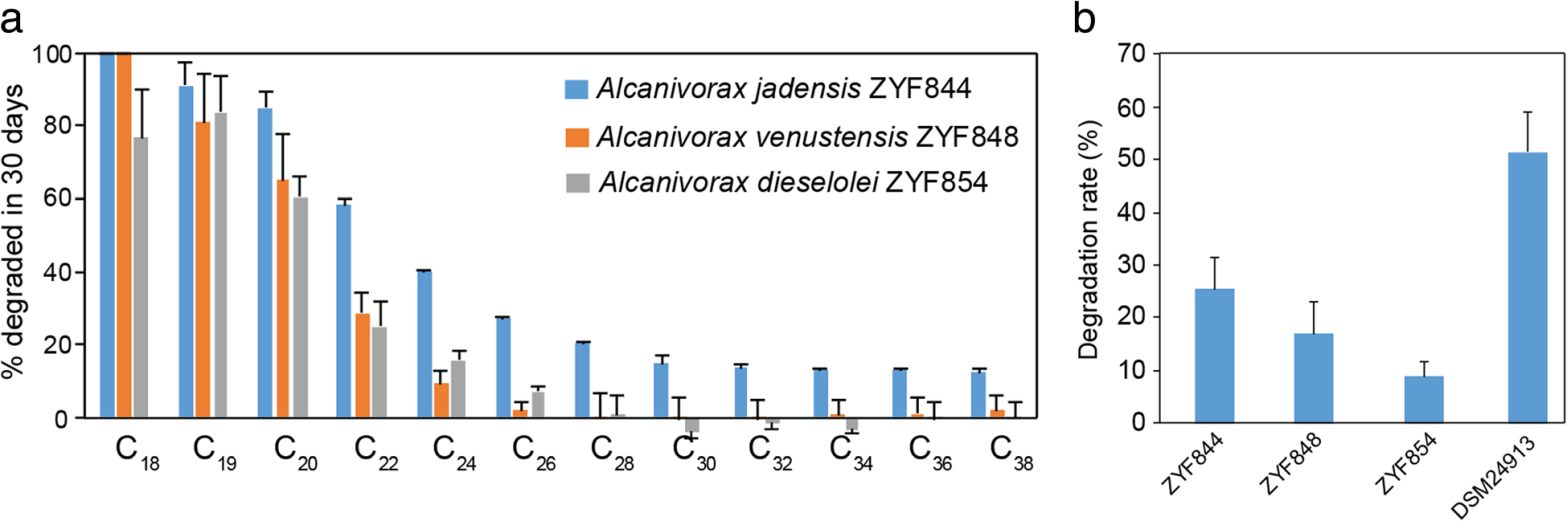
Alkanes are degraded by *Oceanospirillales* isolates. **a** Bacterial degradation rate of C_18–38_ *n*-alkanes at 4 °C, 0.1 MPa for 30 days. **b** *n*-eicosane at 4 °C, 60 MPa for 20 days for *Alcanivorax* strains (ZYF844, ZYF848, ZYF854), and 16 °C, 0.1 MPa for 12 days for *O. marinus* DSM 24913

To complement genetic predictions that NBW bacteria degrade hydrocarbons, we isolated 38 axenic alkane-degrading strains from NBW on alkane-dependent medium. Most isolates were *Oceanospirillales* and mainly affiliated with *Alcanivorax*, including *A. jadensis* ZYF844, *A. venustensis* ZYF848, and *A. dieselolei* ZYF854 (Additional file 1: Figure S13 and Table S6). The three *Alcanivorax* isolates showed 99.6–100% 16S rRNA gene similarity to sequences derived from high-throughput amplicon sequencing, indicating that they represent indigenous NBW species. These three isolates degraded a wide range (C_18_–C_26_) of *n*-alkanes at 4 °C and 0.1 MPa (atmospheric pressure) (Fig. 5a). Notably, they could utilize *n*-eicosane (C_20_) as the sole carbon source at physiologically relevant temperature (4 °C) and pressure (60 MPa) with the strain ZYF844 displaying the highest degradation rate (Fig. 5b).

*O. marinus* DSM 24913 was tested for hydrocarbon degradation activity, since no *Oleibacter* species was isolated from NBW (despite repeated attempts with alkane-dependent media and Marine Broth under 4/16 °C and 0.1/60 MPa) and *O. marinus* is the only cultured species available in this genus. *Oleibacter* MAG 01, the most abundant clade in NBW, demonstrated 99.0% 16S rRNA gene sequence identity to *O. marinus* DSM 24913. Moreover, MAG 01 contained a putative *alkB* and two *almA* genes, which encoded enzymes with > 99.5% amino acid identity to those of *O. marinus* DSM 24913 [12], and up to 34.0% and 49.7% identity to functionally characterized AlkB and AlmA from *A. dieselolei*, respectively [14, 23]. MAG 01 also harbored 11 putative MCP genes (*O. marinus* DSM 24913 possessed 14 MCP genes), with up to 43.9% amino acid identity to the functionally confirmed *A*. *dieselolei* MCP involved in sensing alkanes [14].

Unlike NBW *Alcanivorax* species, *O. marinus* DSM 24913 could not degrade hydrocarbons at 4 °C and 60 MPa, but it displayed a high rate of hydrocarbon degradation in cultures fed on *n*-eicosane at 16 °C and atmospheric pressure (Fig. 5b). This implies that NBW *Oleibacter* species, such as MAG 01, have evolved to withstand high pressure and low temperature typical of hadal conditions. Thus, we propose that the NBW *Oleibacter* species may represent novel and hadal-specific ecotypes despite their high 16S rRNA gene (i.e., 99.0% identity) and genome sequence (98.6% average nucleotide identity) identity to *O. marinus* DSM 24913. A similar phenomenon was shown for *Alteromonas macleodii* species, which are phylogenetically divided into “surface” or “deep” ecotypes (recently a new species *A. mediterranea* was separated [24]), according to seawater depth, despite sharing > 99% 16S rRNA gene similarity [25]. In support of bioinformatic predictions, *n*-alkanes can be utilized as a carbon and/or energy source by all tested NBW *Oceanospirillales* isolates under high pressure and low temperature, and *O. marinus*, representative of the dominant *Oleibacter* genus in NBW under atmospheric pressure at 16 °C.

### Potential alkane accumulation at deeper depths and production in sediments in the Mariana Trench

**Fig. 6:**
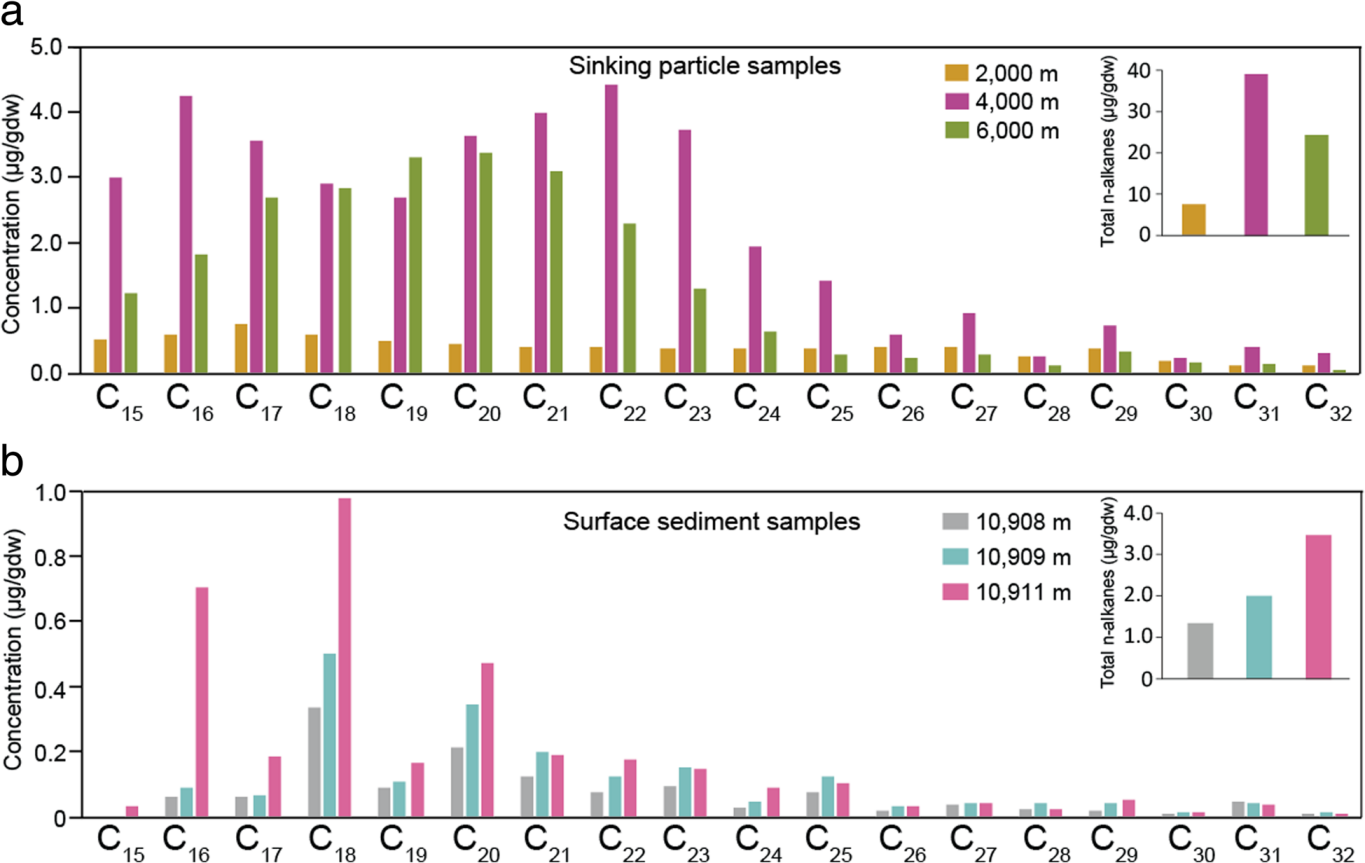
Alkanes accumulate at lower depths. **a** Sinking particle samples. **b** Surface sediment samples. Total *n*-alkane concentrations of each sample are shown in the upper-right corner of **a** and **b**

To study the hydrocarbon sources that may support this population, we investigated alkane concentrations in sinking particle (2000, 4000, and 6000 m; Fig. 6a and Additional file 1: Table S7) and surface sediment samples (Fig. 6b). Total concentrations of *n*-alkanes (in the range of *n*-C_15_ to *n*-C_32_) in sinking particles increased from 2000 (7.2 μg/gdw) to 4000 (39.0 μg/gdw), but then decreased slightly at 6000 m (24.2 μg/gdw). These values are comparable or slightly higher than hydrocarbon concentrations (8.3-37 μg/g; dominated by *n*-alkanes) reported for sinking particles from abyssal waters of the northeastern Pacific Ocean [26]. The unique funnel-shaped geomorphology of the trenches (Additional file 1: Figure S1) promotes the accumulation of particulate organic matter sinking from the surface [27], including possible terrestrial organic matter sources [27], facilitated by lateral transportation from trench rims and slopes that is often triggered by earthquakes [28]. Considering this, we hypothesize that *n*-alkane hydrocarbons maintain high concentrations, or even increase, at depths below 6000 m. Unfortunately, we were unable to analyze alkane concentrations in sinking particle samples at deeper depths, so further work is required to test this hypothesis.

**Fig. 7:**
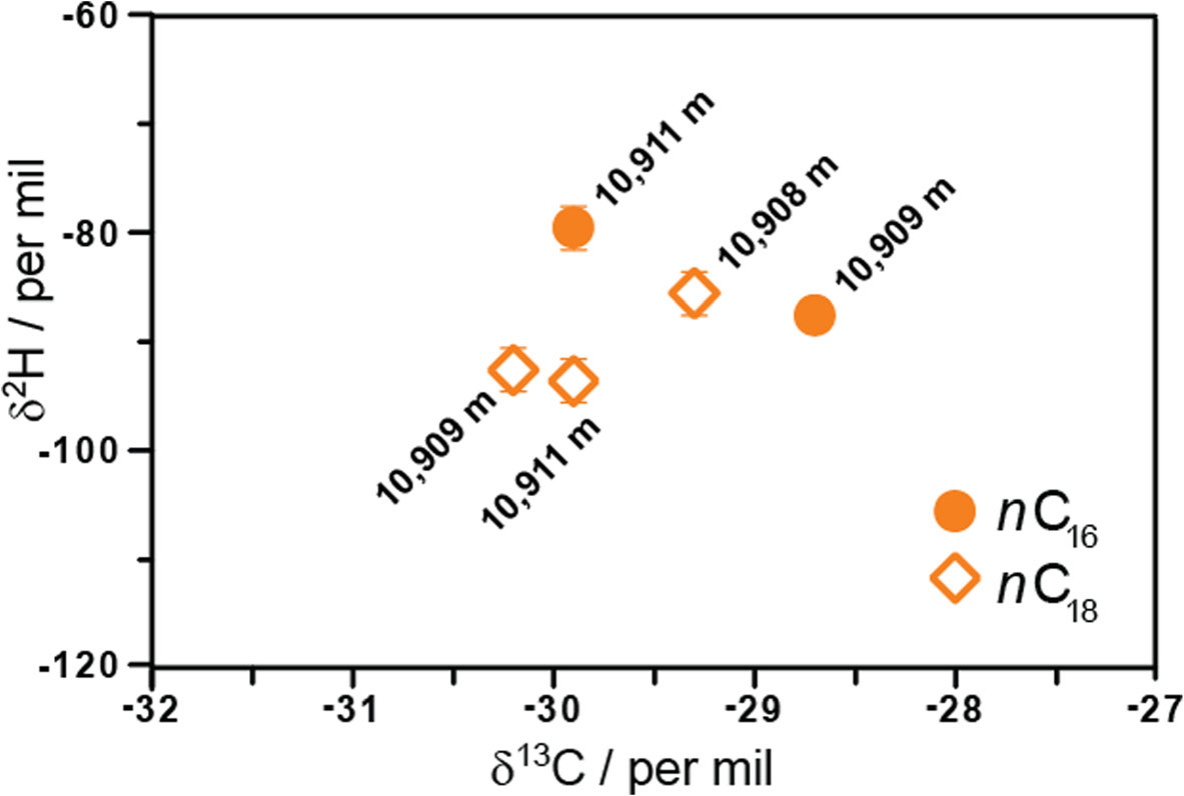
The carbon and hydrogen isotopic compositions of *n*-C_16_ and *n*-C_18_ alkanes from hadal surface sediments. The errors are based on duplicate measurements

To determine the sources of *n*-alkanes in hadal surface sediments at depths of 10,908, 10,909, and 10,911 m (Fig. 6b and Additional file 1: Table S7), we investigated their distribution and concentrations (total *n*-alkane concentration averaged 2.3 μg/gdw). Interestingly, higher concentrations of *n*-C_18_ and *n*-C_20_ (and *n*-C_16_ in one sample) compared to *n*-C_17/19/21_ alkanes (Fig. 6b) were observed. The predominance of even-chain alkanes has been reported previously by other studies, e.g., in sediments from the Black and Mediterranean Seas, Arabian Gulf, and Cariaco Trench [29], and from the Mariana Trench sediments at depths of 4900–7068 m [30]. These were markedly different from those profiles seen in the sinking particle samples, suggesting that even-chain alkanes in hadal surface sediments have a very different source and are likely synthesized in situ and/or released from subsurface sediments. To provide further evidence for the origin of these compounds, we investigated the carbon and hydrogen isotope compositions of *n*-C_16_ and *n*-C_18_ alkanes (Fig. 7). The δ^13^C values of *n*-C_16_ and *n*-C_18_ alkanes ranged from − 30.2 to − 28.7‰, similar to those reported by Guan et al. [30], which are not very source specific as these values fall within the range of isotopic compositions of hydrocarbons derived both from petroleum as well as from autotrophic and heterotrophic organisms [31]. However, more crucially, the δ^2^H values of *n*-C_16_ and *n*-C_18_ alkanes are much more informative. They varied from − 79 to − 93‰, which are higher than those typically assigned to petroleum sources (− 90 to − 150‰ [32]) and fall within the range of lipid/water ^2^H/^1^H fractionations reported for heterotrophic organisms (− 150‰ and higher [33]) assuming the δ^2^H of water is ca. 0‰. These δ^2^H data suggest that *n*-alkanes in the hadal sediments most likely derive from a distinct biological source (e.g., from heterotrophic communities [33]) that results in a smaller hydrogen isotope fractionation. This source differs from that produced by known hydrocarbon-producing marine organisms, predominantly photoautotrophs, which synthesize only odd-C-numbered hydrocarbons [34–36], suggesting that the microbial producers might utilize a different, and still poorly understood biosynthetic pathway.

Given the NBW *Alcanivorax* isolates were more efficient at degrading C_18_–C_20_ compared to > C_22_ alkanes (Fig. 5a), it is possible that these bacteria have evolved to take advantage of these specific hydrocarbons. Thus, this unknown biological source of *n*-C_16_, *n*-C_18_, and *n*-C_20_ hydrocarbons in the surface sediments at the bottom of the trench may contribute, with sinking particle hydrocarbons, to support the observed NBW hydrocarbon-degrading microbial population. Further work, involving turnover rate measurements, is likely required to establish this hypothesis. A similar hydrocarbon cycle is likely present in surface waters, where hydrocarbon-degrading bacteria are supported by alkanes synthesized by cyanobacteria and algae [34]. Currently, it is uncertain if these hydrocarbons are produced by microbes only found in the sediments or whether they are widespread in NBW, since we were unable to study their prevalence in NBW samples and the enzymes synthesizing even-chain alkanes have not been identified.

## Conclusions

In summary, this study demonstrates the highly stratified metabolic potential of the microbial community in the hadal zone of the Mariana Trench. The deepest waters (> 10,000 m) are enriched with alkane-degrading bacteria of *Oceanospirillales* at a proportion higher than observed anywhere else on Earth, which are transcribing alkane-degrading genes. The study raises important questions that warrant further investigation in this unique environment. Which sources of alkanes support microorganisms in the deepest waters on Earth: novel biological alkanes identified in deep hadal sediments or alkanes derived from other sources (e.g., surface water photoautotrophs and/or petroleum), or both? Further, what causes the abrupt and unexpected shift in metabolic potential towards hydrocarbon degradation at depths below 10,000 m and are such shifts common in other deep trench environments?

## Methods

### Sample collection

Seawater at 0 (cast #101, 200 L), 4000 (cast #102, 200 L), 9600 (cast #103, 96 L), 10,400 (cast #104, 96 L), and 10,500 m (cast #105, 96 L) was collected using Niskin bottles from the Challenger Deep of the Mariana Trench (11° 20.605′ N, 142° 19.557′ E, 10,800 m) aboard the R/V *Dong Fang Hong 2* in September 2016. Collection points are shown in Additional file 1: Figure S1 [37]. Water salinity and temperature (Additional file 1: Table S2) were recorded by CTD. For each depth, water samples from three or four Niskin bottles were pooled and filtered onto one membrane. It took 4–5 h to retrieve seawater from the deepest depth (10,500 m). Upon resurfacing, biomass collection was completed within 1 h. Seawater was filtered serially through 3 μm (TSTP, 142 mm, Millipore) and 0.22 μm (GTTP, 142 mm, Millipore) polycarbonate membranes. The communities collected on the 3-μm and 0.22-μm filters were designated as particle-associated and free-living fractions, respectively. All filters were stored in liquid nitrogen onboard and at − 80 °C in the laboratory. Sinking particles were collected at the same location as seawater using time series sediment traps (McLane Research Laboratories, Mark-7) deployed at three depths (2000 m, 4000 m, and 6000 m) from January to September 2016. The surface sediment samples were collected from the Mariana Trench (11° 19.5132′ N, 142° 11.2906′ E) at a depth of ~ 10,910 m during research cruise TS-03 in January 2017 using the lander “TianYaHao” aboard the R/V *TAN SUO YI HAO* (see details in Additional file 2: Supplementary method).

### DNA and RNA extraction

DNA was extracted from water samples of both fractions at all five depths as previously described [38]. Briefly, biomass was washed from half of each filter using 15-ml extraction buffer (100 mM Tris-HCl [pH 8.0], 100 mM sodium EDTA [pH 8.0], 100 mM sodium phosphate [pH 8.0], 1.5 M NaCl, 1% CTAB) and then centrifuged at 5000×*g* for 20 min at room temperature. The concentrated biomass was ground within liquid nitrogen. Proteinase K and SDS were added in sequence and incubated at 37 °C and 65 °C, respectively, followed by phenol-chloroform extraction. DNA was precipitated with 0.6 volume of isopropanol, washed with 70% ethanol, air dried, and dissolved in TE buffer. The concentration and integrity of genomic DNA were analyzed by Qubit fluorometer and agarose gel electrophoresis, respectively. RNA was extracted using the Trizol reagent and cleaned using the Direct-zol RNA MiniPrep kit. DNA removal was performed using the TURBO DNA-*free* kit. Successful DNA removal was confirmed by 16S rRNA gene PCR. cDNA was generated using the Promega M-MLV reverse transcriptase.

### Metagenomic sequencing, 16S rRNA gene high-throughput sequencing, and clone library construction

Nine DNA samples (each greater than 1.5 μg), except for the particle-associated fraction of 9600 m due to an insufficient quantity of DNA, were sent to BGI (BGI, Shenzhen, China) for metagenomic sequencing. Libraries were prepared without any amplification step for each sample. Metagenomic shotgun sequencing was performed on the Illumina HiSeq X-Ten platform, with 2 × 150-bp paired-end reads. Primers 515F and 806R (Additional file 1: Table S8) were used to amplify the V4 region of the 16S rRNA gene, and DNA extracted from three membranes were treated as triplicate. Sequencing was performed on the Illumina Miseq PE300 platform (MiSeq Reagent Kit v3) at Majorbio BiFo-Pharm Technology Co., Ltd., Shanghai, China. Sequences were processed with the pipeline of UPARSE [39]. Operational taxonomic units (OTUs) were clustered at a 97% similarity level and taxonomically assigned against the SILVA SSU reference database (release 132). To examine the phylogenetic relationship between bacterial isolates and environmental sequences, a clone library was constructed using DNA from the FL10500 sample with primers B8F and B1510R (Additional file 1: Table S8). PCR products were inserted into pUCm-T vectors and cloned into *Escherichia coli* JM109. Positive clones were checked with PCR using primers M13F/R. Correct clones were sequenced at BGI (Qingdao, China) and taxonomically assigned using the EzTaxon-e server (https://www.ezbiocloud.net).

### Quantitative PCR and reverse transcription qPCR

The abundance of bacterial 16S rRNA genes was quantified using quantitative PCR (qPCR) with primers Eub338F and Eub518R (Additional file 1: Table S8). Genomic DNA extracted from 10,500 m was used as a template to amplify 16S rRNA genes. PCR products were purified using a Gel Extraction Kit (Omega), ligated with pUCm-T vector, and then transformed into competent *E. coli* JM109 cells for sequencing. The sequence of target genes was submitted to the EzTaxon-e server (https://www.ezbiocloud.net) and identified as *Ulvibacter* sp. (*Flavobacteria*). The confirmed plasmid was extracted using a Plasmid Mini Kit (Omega) and digested with restriction enzyme XhoI. The linear plasmid was purified using a Gel Extraction Kit (Omega) and was serially diluted to construct standard curves (concentration ranged from 2.91 × 10^2^ to 2.91 × 10^7^ copies/L). The reverse transcription qPCR (RT-qPCR) standards for the *alkB* and *almA* gene (Additional file 1: Table S8) were prepared as detailed in the above description for the 16S rRNA gene. qPCR was performed on the StepOnePlus Real-Time PCR System based on SYBR Green I.

### Assembly and binning

The reads that contain more than 10% of undefined bases and more than 40% of low-quality bases, and had a length of more than 15 bases matching to the adapters were removed. After filtering, clean data ranging in size from 13.67 to 16.54 Gb was obtained from each sample, and the average Q30 of each sample was above 93%, indicating that the trimmed dataset was of high quality. High-quality reads were assembled using SOAP*denovo*2 [40] with a kmer size of 49. Contigs shorter than 500 bp were discarded. Gene prediction was performed using MetaGeneMark [41] with default parameters (http://exon.gatech.edu/GeneMark/meta_gmhmmp.cgi).

Metagenomic binning was performed on four bottom water samples (FL10400, PA10400, FL10500, and PA10500). The initial paired-end reads from these four samples were co-assembled using IDBA-UD [42]. Subsequently, these reads were mapped to the final contigs with BWA [43]. Contigs were grouped into MAGs based on empirical probabilistic distances of genome abundance and tetranucleotide frequency by using program MetaBAT2 [44]. Completeness and contamination of MAGs were assessed using CheckM [45], and MAGs with a completeness above 80% and contamination lower than 10% were considered for further analysis. PhyloPhlAn [46] was used to determine the phylogeny of MAGs, following by the average nucleotide identity (ANI) calculated to further confirm the taxonomy. The relative abundance of each MAG was calculated by mapping each of them to the raw reads. Gene calling of the MAGs was carried out using the RAST server [47]. All the predicted genes were searched against the SEED subsystems, COG (ftp://ftp.ncbi.nlm.nih.gov/pub/COG/), and KEGG (http://www.kegg.jp/kegg/) databases.

### Taxonomic assignment and functional characterization

Genes were clustered to remove redundant sequences using CD-Hit [48] at the 95% identity and 90% coverage. The gene annotation was done using BLASTp with an *e*-value cutoff of 10^−5^ against KEGG, COG, SEED, and NCBI-nr (released August 2016) databases for functional and taxonomic analysis, and only the best hits were retained. Taxonomy assignment was performed in MEGAN software [49] (version 4.6) based on the BLAST results of nr database using the lowest common ancestor (LCA) algorithm. To verify the taxonomic assignment results, metagenomic reads were mapped onto sequences of the SILVA SSU Ref NR99 database (release 132) using the BBMap package (http://sourceforge.net/projects/bbmap/) with 97% identity. The sequence coverage, which is the total length of mapped reads divided by the reference sequence length, was determined. The relative abundance of each taxon was calculated from the sequence coverage of each taxon (*E*) and the total sequence coverage (*T*) as *E*/*T* × 100%. Gene abundance was normalized by the number of the mapped reads of the target gene to the gene length. The taxa that were significantly enriched in the four NBW samples were described. Averaged abundances across the four NBW samples were provided. Standard deviations were calculated to account for within-group differences.

The marker genes (Additional file 3) for the major known types of aliphatic hydrocarbon-degradation enzymes, i.e., methane monooxygenase (MMO), gaseous short-chain alkane (C_2_–C_4_) monooxygenase, medium-chain alkane degradation enzymes (AlkB and cytochrome P450 monooxygenases, degrading C_5_–C_26_), long-chain alkane monooxygenase (represented by AlmA, degrading C_14_–C_36_), and other relevant genes potentially involved in hydrocarbon degradation were searched against the metagenomic data using BLASTp with an *e*-value cutoff of 10^−5^. An identity value of 40% was used for AlkB, cytochrome P450, and AlmA to increase the possibility of capturing the real functional proteins. All MMO sequences retrieved from the BLASTp results were further annotated in the NCBI-nr database, since they all showed low identity (< 42.9%) compared to marker genes. Only those sequences with top hits to methane monooxygenase in the NCBI-nr database were finally kept. Sequences of *alkB* and cytochrome P450 were retrieved from a functional gene pipeline (http://fungene.cme.msu.edu/). Short-chain (C2-C4) hydrocarbon monooxygenase and long-chain alkane monooxygenase were retrieved from NCBI according to Li et al. [50] and Wang and Shao [14], respectively. Reference sequences of methane monooxygenase were downloaded from the RefSeq database at NCBI. Genes involved in three intermediates (catechol, protocatechuate, and gentisate) of the aromatic degradation pathway were retrieved from the GeoChip 4.6 (http://www.ou.edu/ieg/tools) and RefSeq database at NCBI (Additional file 3). Statistics where applied were examined with the Wilcox ranking test.

### Bacterial cultivation and *n*-alkane degradation

Water samples (10,400 m) were enriched using MMC medium (24 g NaCl, 7.0 g MgSO_4_·7H_2_O, 1 g NH_4_NO_3_, 0.7 g KCl, 2.0 g KH_2_PO_4_, 3.0 g Na_2_HPO_4_, and 10 ml of a trace element solution, pH 7.5) with hexadecane as a sole carbon source and cultivated at 10 °C, atmospheric pressure on a rotary shaker (170 rpm). A total of 38 bacterial strains were obtained. Taxonomic assignment was determined by using the EzBioCloud server (http://www.ezbiocloud.net/). Three *Alcanivorax* strains (*A*. *jadensis* ZYF844, *A. venustensis* ZYF848, and *A. dieselolei* ZYF854) were chosen for *n*-alkane degradation tests. Incubations were performed in 50 ml of MMC medium supplemented with 10 μl of alkane mixture at 4 °C, atmospheric pressure for 30 days. After confirmation that these species can utilize alkanes at low temperature, we further tested whether they could degrade *n*-alkanes at high pressure. The strains were incubated in 5 ml of MMC medium supplemented with 5 mg of *n*-eicosane at 4 °C, 60 MPa for 20 days. High pressure incubations were conducted in stainless steel reactors (380 ml, maximum pressure 60 MPa; Nantong Feiyu Oil Science and Technology Exploitation, China). Pressure was delivered by water using a manual pump. Once the incubation was finished, the remaining alkanes were extracted immediately using dichloromethane and analyzed using an Agilent 7890B Gas Chromatography (GC) with an FID detector. *Oleibacter marinus* DSM 24913 (= NBRC 105760) bought from the Marine Culture Collection of China was also tested for *n*-alkane degradation at 4 °C, 60 MPa, but demonstrated poor growth. Thus, this strain was incubated at 16 °C, atmospheric pressure with *n*-eicosane as a sole carbon source (see details in Additional file 2: Supplementary method).

### Analysis of *n*-alkanes

The remaining alkanes in 50- or 5-ml bacterial cultures were extracted using 10 or 2 ml of dichloromethane and analyzed using an Agilent 7890B Gas Chromatograph (GC) with an FID detector. Two further rounds of extraction were performed. A deuterated alkane (C_24_D_50_) was used as an internal recovery standard (see details in Additional file 2: Supplementary method). The *n*-alkane analysis procedure for sinking particle and surface sediment samples was the same as that of the bacterial cultures except for extraction. Sinking particles and surface sediment samples were first freeze-dried and ground using a mortar and pestle. In order to obtain sufficient *n*-alkane yields from sinking particles at each depth, we combined the samples collected at nine time points (from January to September). Alkanes were extracted from 700 mg of sinking particles (for each depth) and 5.0 g surface sediments using 30 ml dichloromethane/methanol (9:1, *v*/*v*) for 10 min. This procedure was repeated four times and the extracts were combined. Dichloromethane and hexane without sample were used as a blank. All the extraction process was conducted in a clean room, and all the glassware were baked at 550 °C for 12 h to remove any organic matter. The alkanes were not analyzed in the original water samples since we were not expecting to find such a large proportion of hydrocarbon degraders below 10,000 m.

### Analysis of carbon and hydrogen isotopic compositions of *n*-alkanes

Once isolated, biomarker *n*-alkanes were analyzed first by gas chromatography (Thermo Scientific Trace 1310) with a flame ionization detector (GC-FID) fitted to a 30-m TG-5SilMS (0.25 mm × 0.25 μm) fused silica column. Sample aliquots of 1 μl were injected by programmed temperature vaporizing (PTV) injector at 320 °C through a split/splitless silcosteel inlet liner. The chromatograph oven was ramped from 60 °C (1 min) to 320 °C (20 min) at 6 °C min^−1^. Helium was used as a carrier at 2 ml min^−1^, and the detector was set to a constant temperature of 320 °C. Stable isotope measurements of biomarker *n*-alkanes were measured by gas chromatography combustion (δ^13^C) or pyrolysis (δ^2^H) isotope ratio monitoring mass spectrometry (GC-C-irMS or GC-pyr-irMS, respectively) with a Thermo Scientific Trace 1310 GC with split/splitless injector and TriPlus RSH liquid autosampler coupled to a Delta V Plus MS (see details in Additional file 2: Supplementary method).

## Additional files

Additional file 1: Supplementary figures and tables (DOCX 3821 kb)

Additional file 2: Supplementary methods (DOCX 33 kb)

Additional file 3: Accession numbers of protein sequences used as query sequence against the metagenomic datasets in this study (XLSX 12 kb)
