# Supplementary material for: Proliferation of hydrocarbon-degrading microbes at the bottom of the Mariana Trench
Source: Microbiome. 2019 Apr 12;7:47. doi: 10.1186/s40168-019-0652-3 (PMC6460516; doi:10.1186/s40168-019-0652-3)
Supplement: Supplementary file 1 — Supplementary figures and tables (DOCX 3821 kb) [file 40168_2019_652_MOESM1_ESM.docx]

**Additional file 1**

**Proliferation of hydrocarbon degrading microbes at the bottom of the Mariana Trench**

Jiwen Liu^1,2†^, Yanfen Zheng^1†^, Heyu Lin^1^, Xuchen Wang^3^, Meng Li^4^, Yang Liu^4^, Meng Yu^3^, Meixun Zhao^2,3^, Nikolai Pedentchouk^5^, David J. Lea-Smith^6^, Jonathan D. Todd^6^, Clayton R. Magill^7^, Wei-Jia Zhang^8^, Shun Zhou^1^, Delei Song^1^, Haohui Zhong^1^, Yu Xin^2,3^, Min Yu^1,2^, Jiwei Tian^9,10*^, Xiao-Hua Zhang^1,2*^

Corresponding author: Xiao-Hua Zhang

Email: [xhzhang@ouc.edu.cn](mailto:xhzhang@ouc.edu.cn)

**Supplementary Figures**


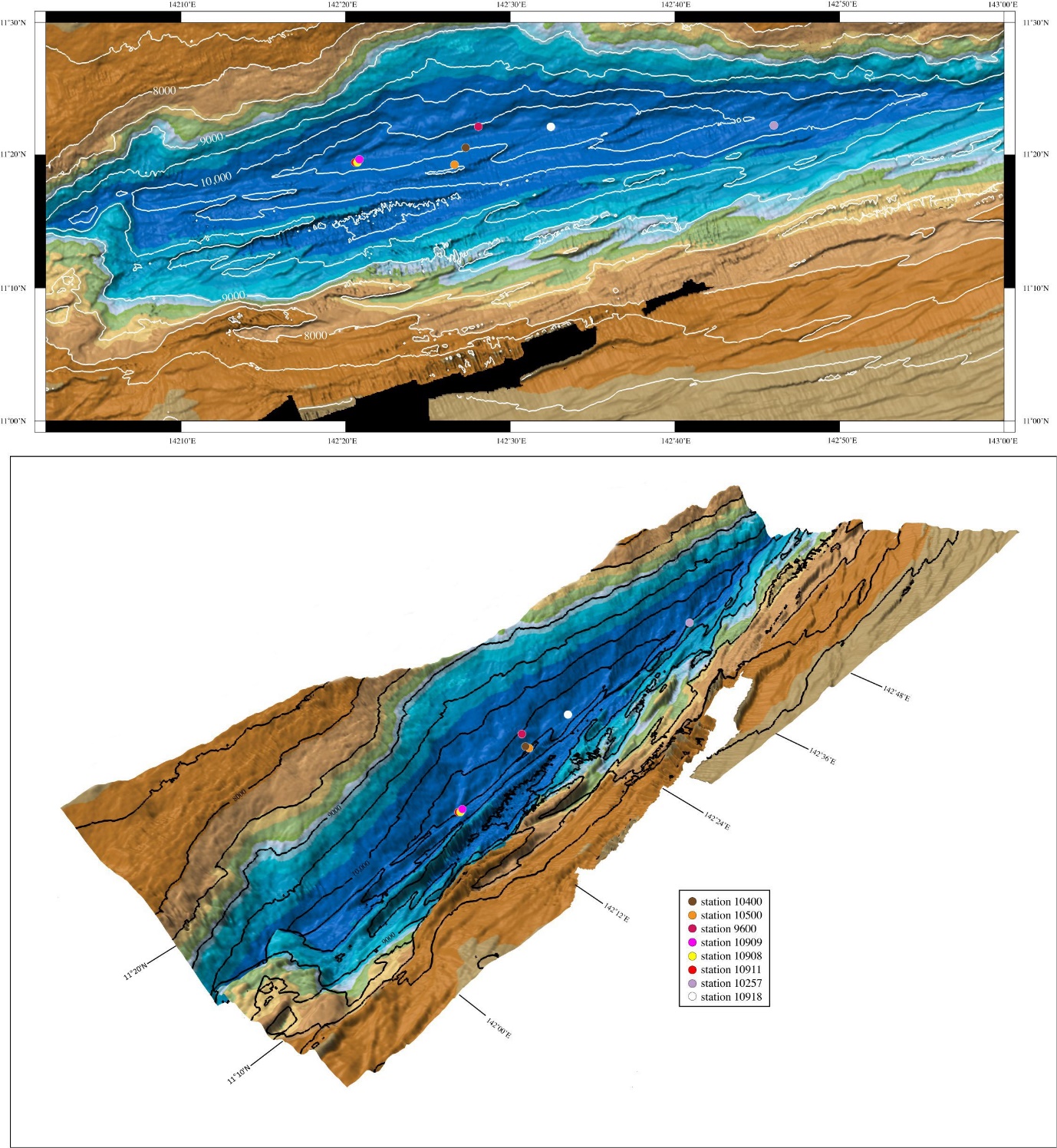


**Figure S1. Map (top) and perspective (bottom) view of multibeam bathymetry of the Challenger Deep area of the Mariana Trench.** Isobaths are at 500 m intervals. Illumination azimuth 315°, elevation 33° for both views; Vertical exaggeration is 5x for the perspective view. Indicated is the location where the seawater (9,600, 10,400, 10,500 m) and sediment (10,908, 10,909, 10,911 m) samples were collected for community analysis in this study and the ones by Nunoura *et al.* (10,257 m) [1] and Tarn *et al.* (10,918 m) [2].


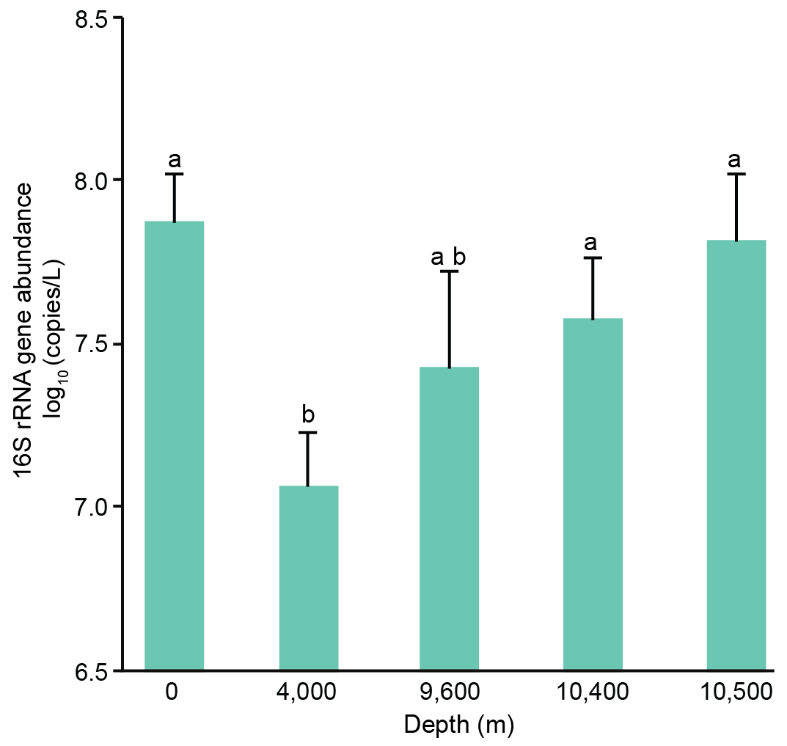


**Figure S2. Depth profiles of the abundance of 16S rRNA gene as determined by qPCR analysis.** The 16S rRNA gene copy number is a summation of the free-living and particle-associated fractions. Increased 16S rRNA gene copy number in bottom water compared to 4,000 m might be due to increased *Gammaproteobacteria* populations, which harbor 5.7 copies of 16S rRNA gene on average, far higher than other *Proteobacteria* [3]. The bars with different letters (i.e., a and b) above indicate that there are significant differences among different depths (*P* < 0.05). Results are from triplicate experiments.


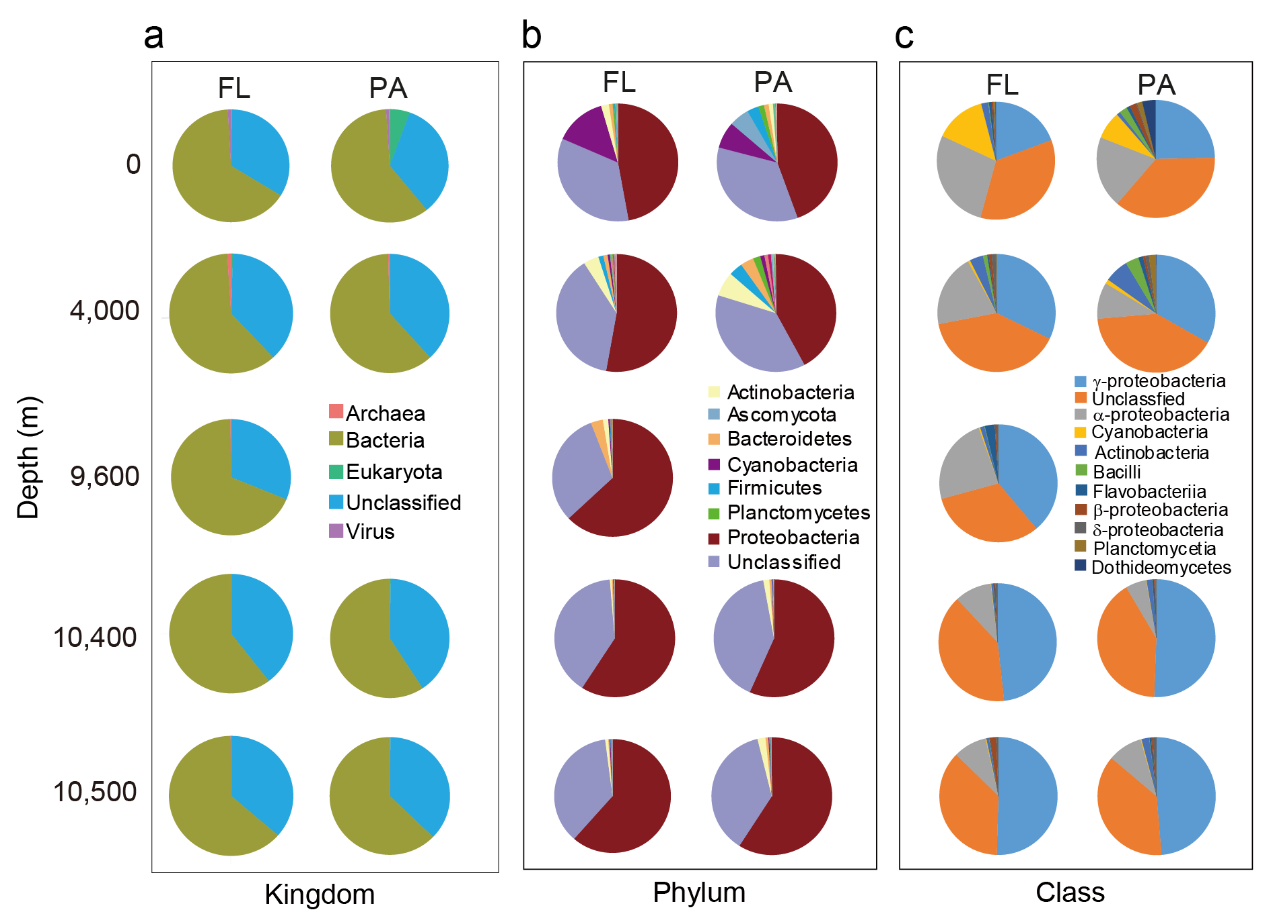


**Figure S3. Microbial taxonomic profiles vary throughout water column in the Challenger Deep at the**: **a** Kingdom. **b** phylum. **c** class level. The analysis is based on the metagenomic data annotation against NCBI-nr database. FL, free-living; PA, particle-associated.


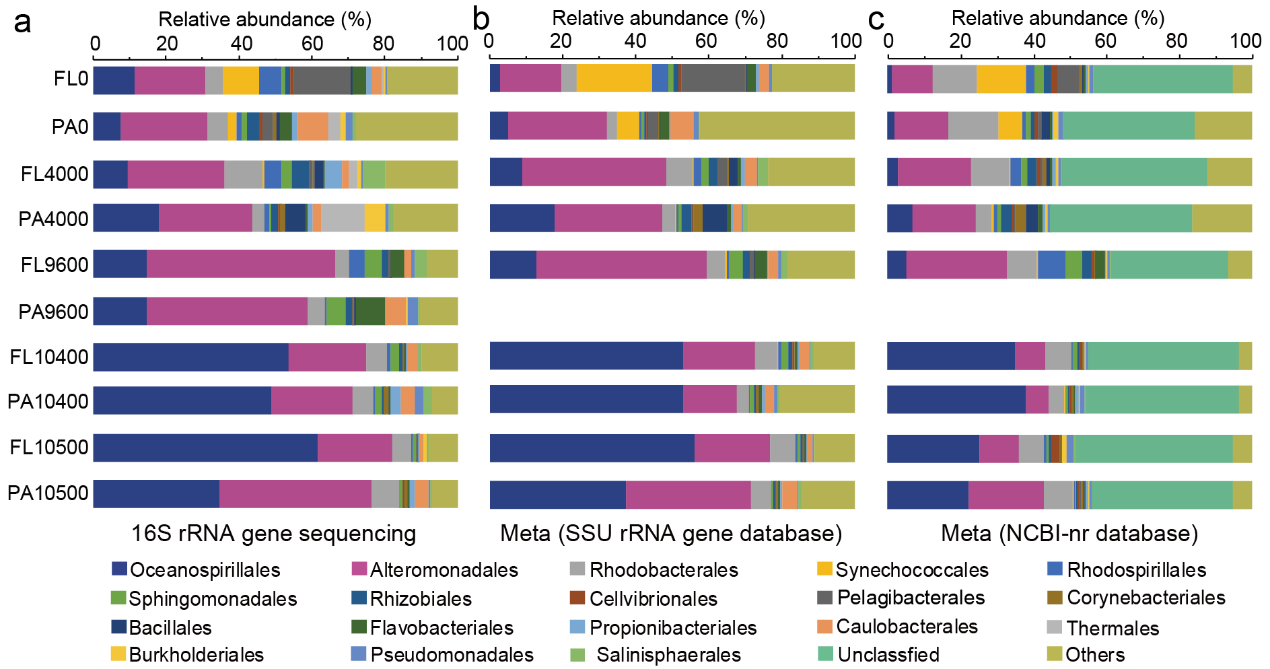


**Figure S4. Depth profiles of the microbial community compositions at the order level.** **a** 16S rRNA gene amplicon sequencing (triplicate). **b** Metagenomic data against SILVA SSU rRNA gene database. **c** Metagenomic data against NCBI-nr database. Colors indicate different bacterial orders as indicated.


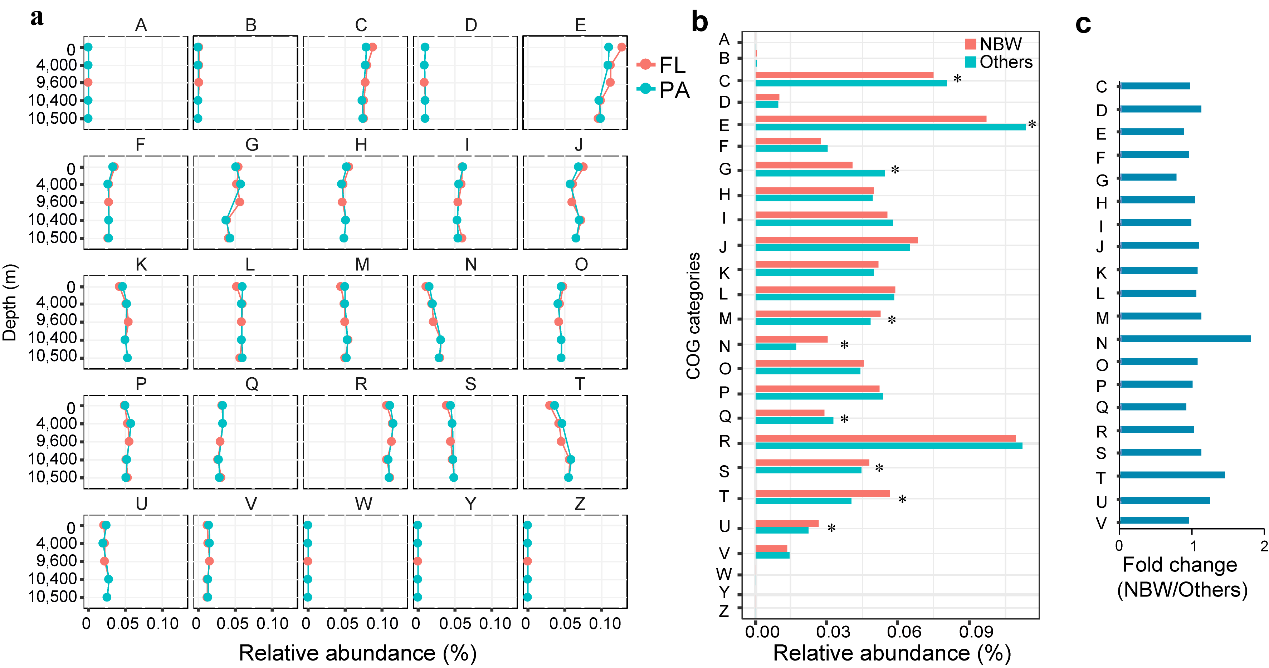


**Figure S5. Relative abundances of genes based on COG functional categories between NBW and others.** **a** Depth profiles of all categories. **b** Categories significantly enriched are marked with a star. **c** Fold change in relative abundance between NBW and other samples. Categories A, B, W, Y and Z were not included in the latter two panels due to their low abundance. NBW, near bottom water. COG categories are as follows: A, RNA processing and modification; B, Chromatin structure and dynamics; C, Energy production and conversion; D, Cell cycle control, cell division, chromosome partitioning; E, Amino acid transport and metabolism; F, Nucleotide transport and metabolism; G, Carbohydrate transport and metabolism; H, Coenzyme transport and metabolism; I, Lipid transport and metabolism; J, Translation, ribosomal structure and biogenesis; K, Transcription; L, Replication, recombination and repair; M, Cell wall/membrane/envelope biogenesis; N, Cell motility; O, Posttranslational modification, protein turnover, chaperones; P, Inorganic ion transport and metabolism; Q, Secondary metabolites biosynthesis, transport and catabolism; R, General function prediction only; S, Function unknown; T, Signal transduction mechanisms; U, Intracellular trafficking, secretion, and vesicular transport; V, Defense mechanisms; W, Extracellular structures; Y, Nuclear structure; Z, Cytoskeleton.


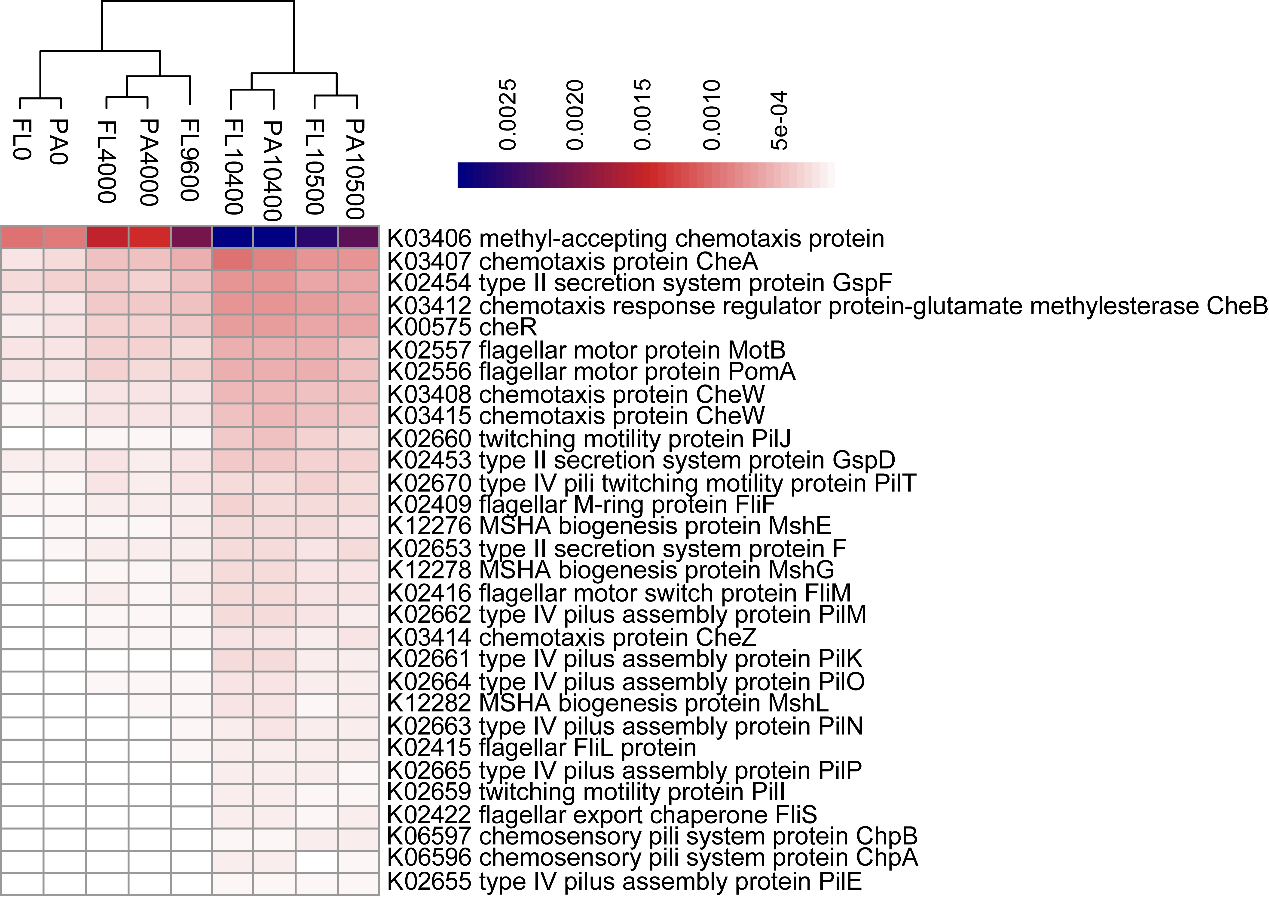


**Figure S6. Heat map showing the genes affiliated with cell motility (COG N) dominant in the four near bottom water (NBW) samples.** Thirty genes with the highest fold changes and relative abundances are shown. They are all significantly represented in NBW (*P* < 0.05).


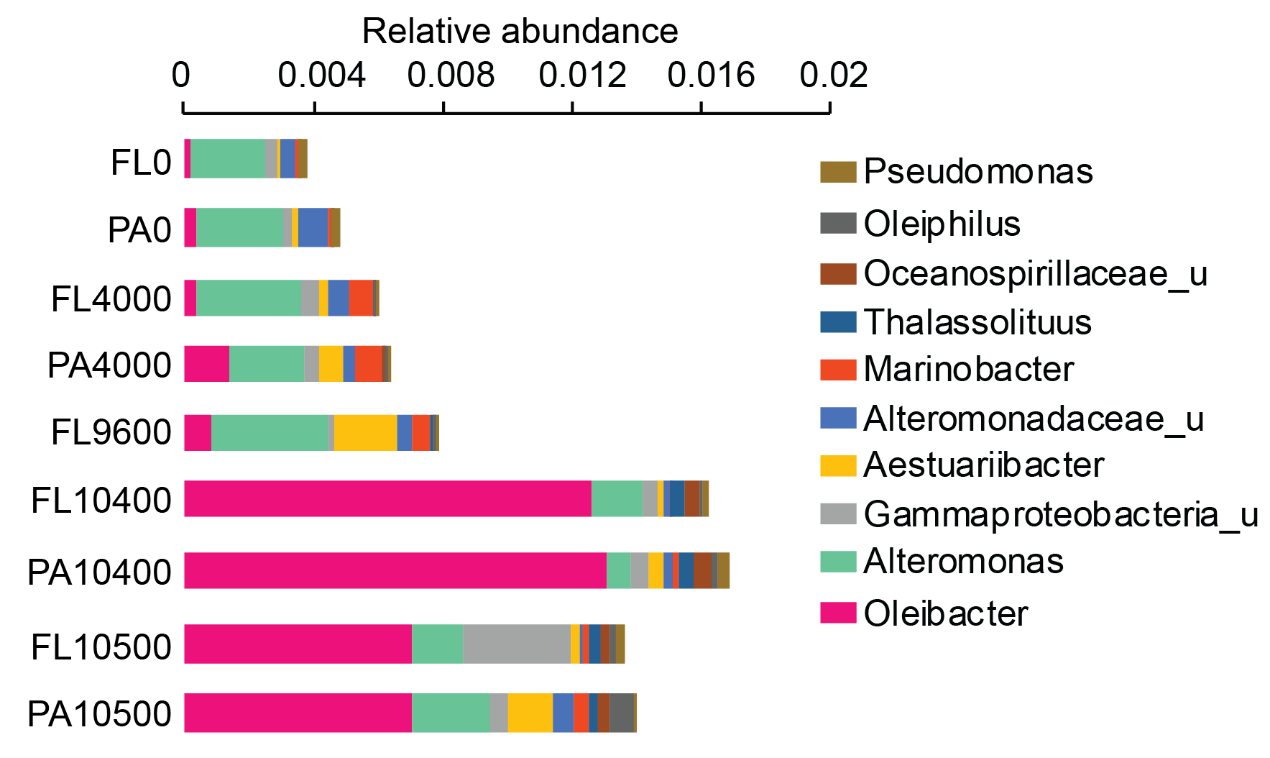


**Figure S7. Taxonomy assignment of relevant COG N (cell motility).** u: unclassified. Colors indicate different bacterial genera as indicated.


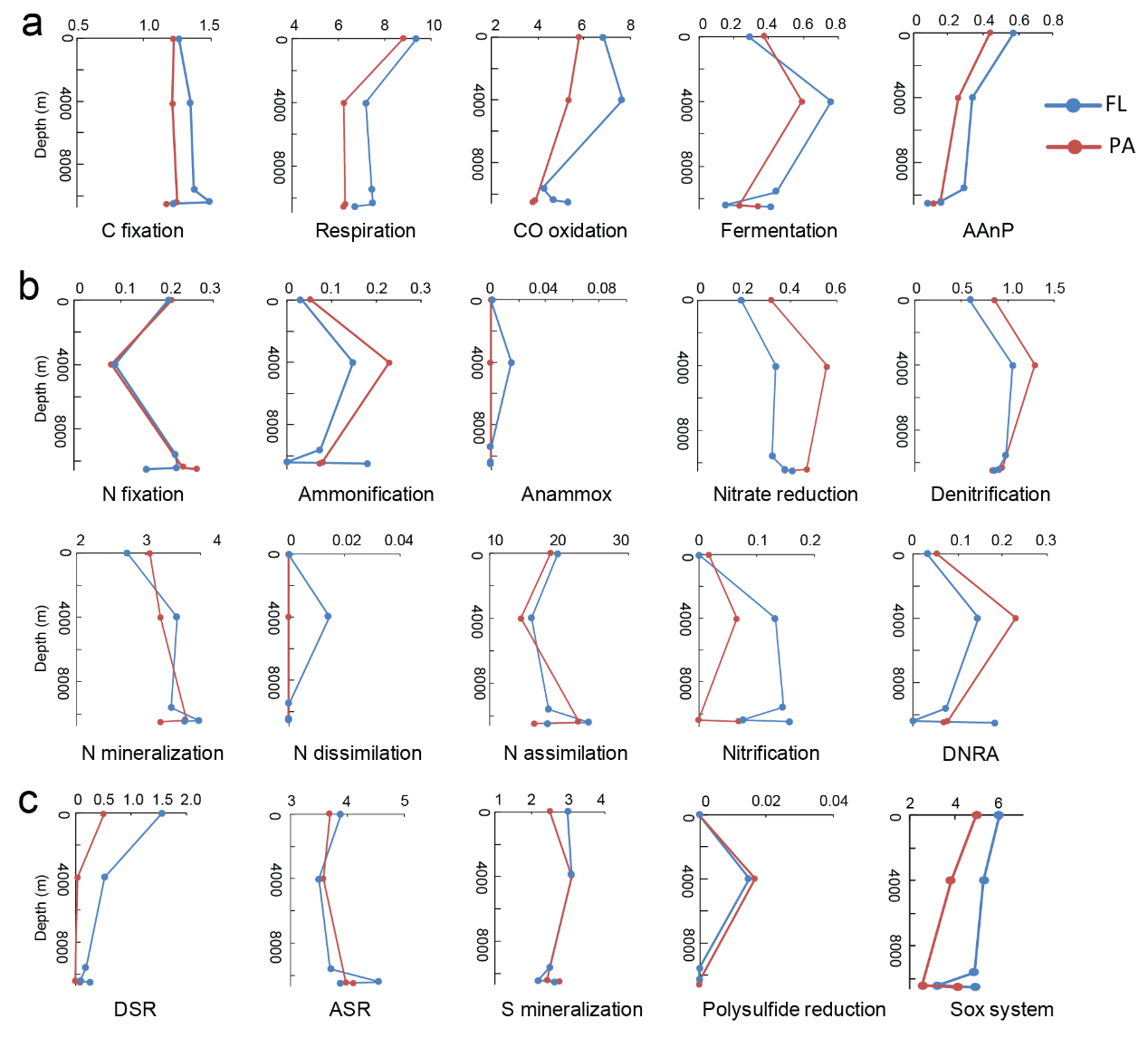


**Figure S8. Depth profiles of the relative abundance of genes related to carbon, nitrogen and sulfur cycling.** **a** Carbon cycling related genes. **b** Nitrogen cycling related genes. **c** Sulfur cycling related genes. The x-axis represents counts of marker genes normalized to 10,000 genes and the y-axis shows sample depths (m). The free-living (FL) and particle-associated (PA) fractions are indicated in blue and red, respectively. For the same pathway or enzyme complex, a summation of the relative abundance of all marker genes involved is shown. AAnP, aerobic anoxygenic photosynthesis; DNRA, dissimilatory nitrate reduction to ammonia; DSR, dissimilatory sulfate reduction; ASR, assimilatory sulfate reduction. Sox, sulfur oxidation.


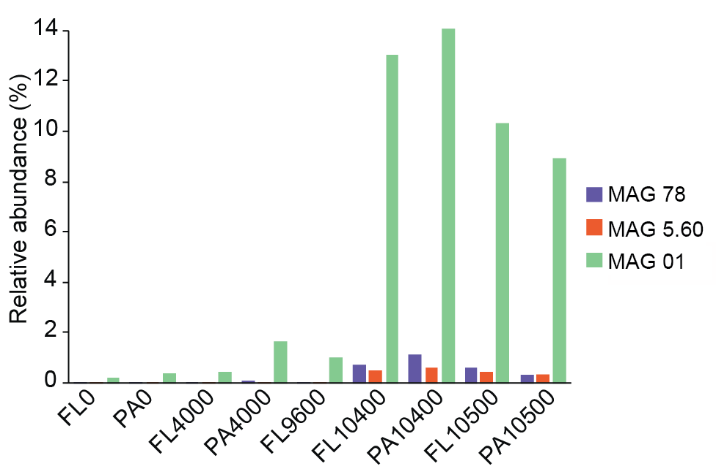


**Figure S9. The relative abundance of the three *Oleibacter* MAGs at different depths.** MAGs and the single-copy gene *recA* were mapped to raw reads of metagenomic data. The relative abundance of each MAG was normalised to that of *recA*.

**
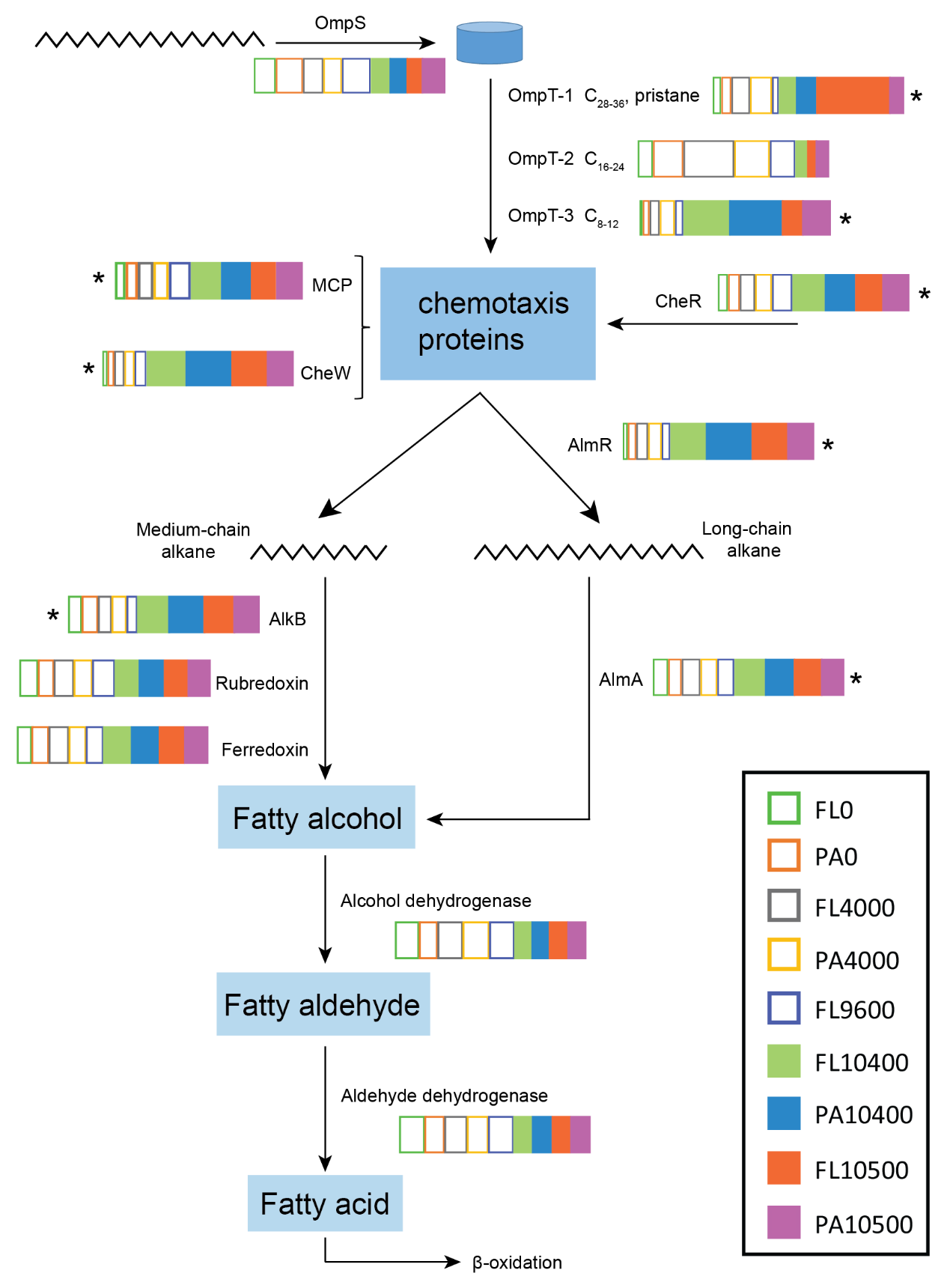
**

**Figure S10. Relative abundance of genes involved in each step of alkane degradation processes.** Gene sequences in the metagenomics data involved in alkane degradation pathways were retrieved by BLASTp against genes reported by Wang and Shao [4]. Bars with filled and unfilled colors represent near bottom water (NBW) and other water layers, respectively. The asterisk indicates that the relative abundance of genes in the NBW are significantly higher (*P* < 0.05) than in the other layers. OmpS: outer membrane protein; OmpT: long-chain fatty acid transporter protein; MCP: methyl-accepting chemotaxis protein; CheW: chemotaxis protein; CheR: chemotaxis protein methyltransferase; AlmR: regulatory protein of LC-alkane metabolism. AlmA: long-chain alkane monooxygenase; AlkB: alkane 1-monooxygenase.


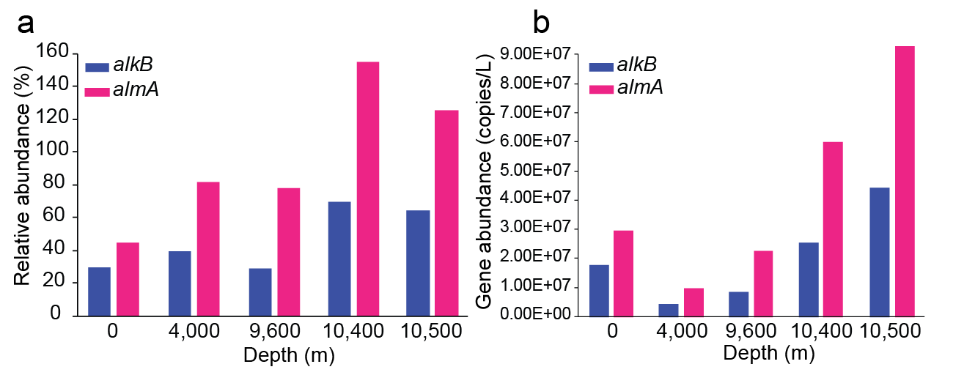


**Figure S11. The relative and absolute abundance of *alkB* and *almA* at different depths. a** The proportion of bacterial cells containing genes *alkB* or *almA*. **b** The quantity of *alkB* and *almA*. The values in panel **a** are normalised to the single-copy gene *recA*. The value in panel **b** was obtained by multiplication of the total bacterial abundance derived from qPCR of the 16S rRNA gene and the proportion of bacterial cells carrying the respective marker gene.


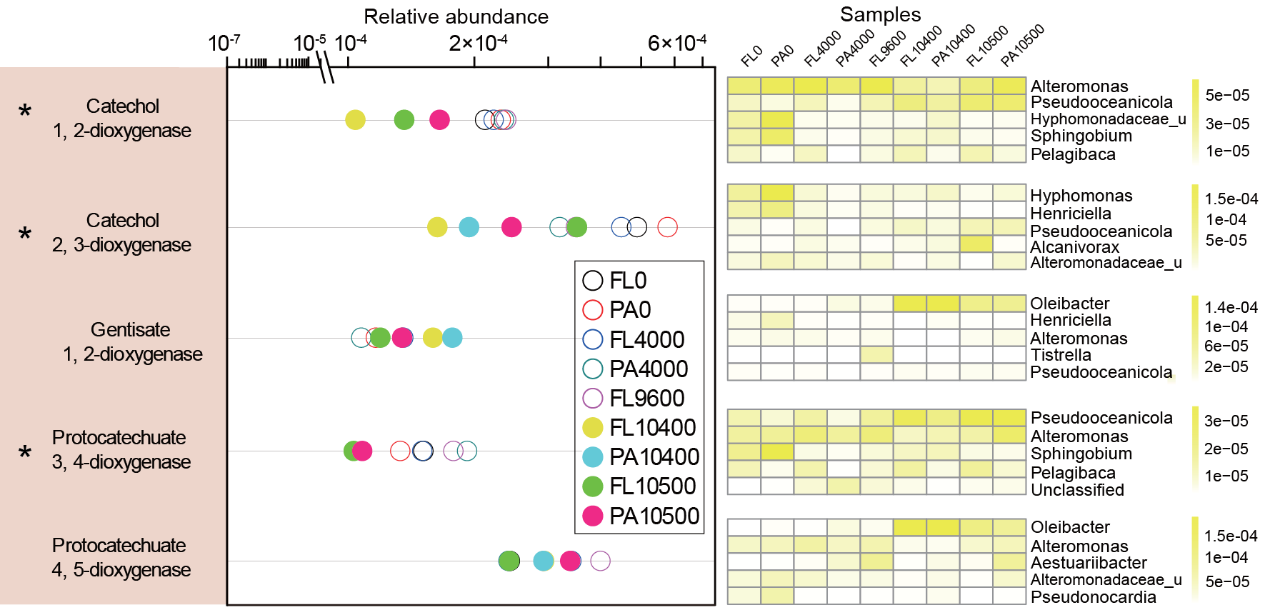


**Figure S12. Shifts in relative abundance of genes associated with aromatic degradation with depth.** The top five affiliated genera of each gene are shown in the heat map (right). Asterisks indicate statistically significant difference in gene abundance between the two zones.


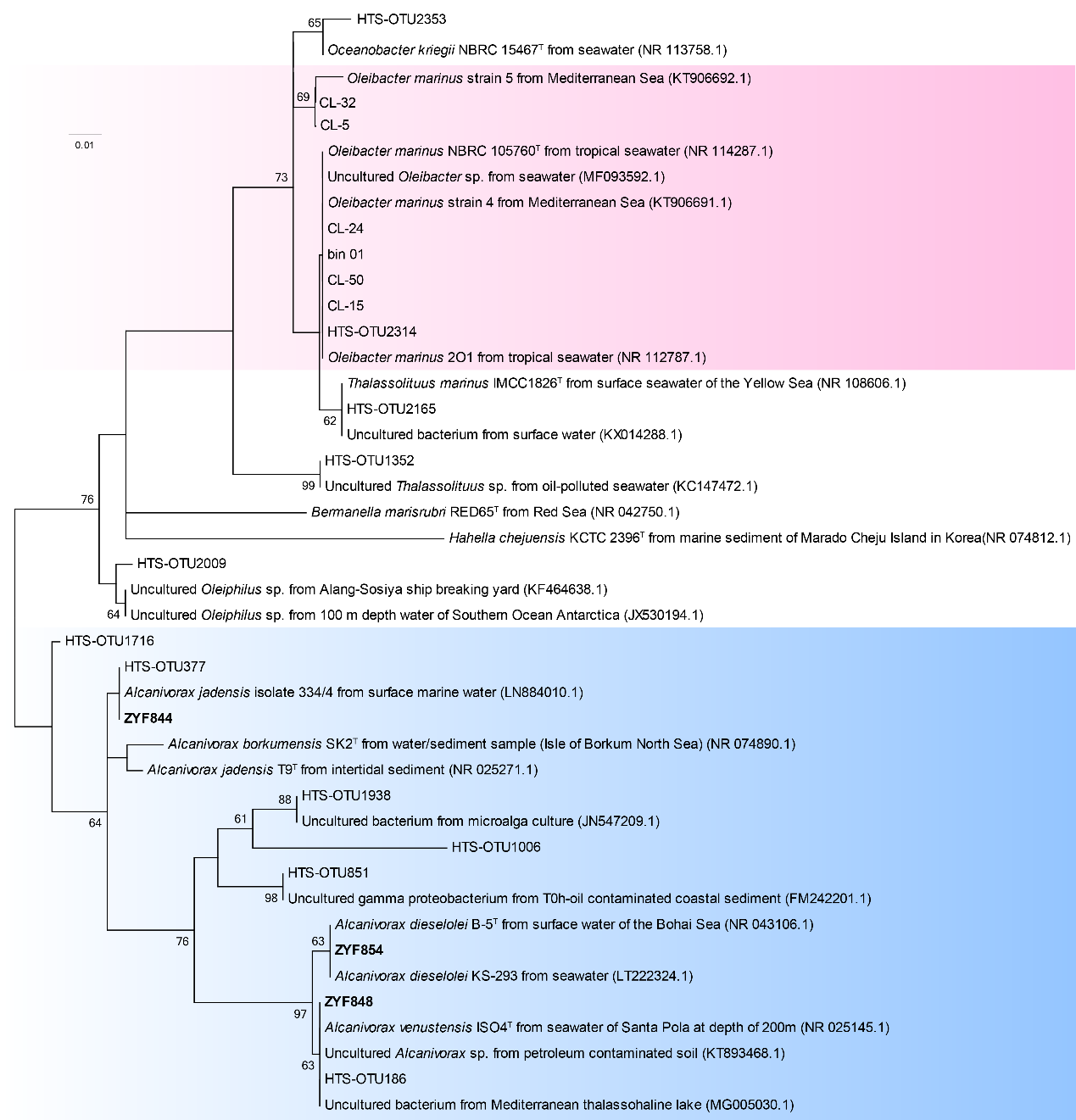


**Figure S13. The neighbour-joining phylogenetic tree constructed with the 16S rRNA gene sequences obtained from the FL10500 sample clone library (CL), high-throughput amplicon sequencing (HTS) derived OTUs from NBW, and the reference strains.** ZYF844, 854 and 848 are the three *Alcanivorax* strains tested for hydrocarbon degrading capability.

**Supplementary Tables**

Table S1. General features of the metagenomic data

| Sample | Clean data (Gb) | Number of contigs | Number of contigs  > 1kb | Assembly length (bp) | Longest contig (bp) | N50 (bp) | Average G+C contents (%) | Mapping rate (%) |
| --- | --- | --- | --- | --- | --- | --- | --- | --- |
| FL0 | 14.79 | 401,248 | 134,616 | 533,807,522 | 257,326 | 1,580 | 50.32 | 42.92 |
| PA0 | 16.54 | 189,022 | 49,245 | 208,617,148 | 365,904 | 1,138 | 52.98 | 19.98 |
| FL4000 | 16.10 | 392,156 | 155,386 | 666,744,620 | 512,957 | 2,639 | 56.35 | 56 |
| PA4000 | 16.34 | 423,262 | 160,428 | 717,161,825 | 566,681 | 2,734 | 57.02 | 53.58 |
| FL9600 | 14.99 | 333,595 | 119,235 | 568,373,836 | 428,605 | 2,761 | 54.58 | 63.21 |
| FL10400 | 14.61 | 273,226 | 95,748 | 396,765,532 | 467,516 | 1,837 | 55.08 | 53.67 |
| PA10400 | 14.61 | 218,086 | 84,607 | 373,094,977 | 437,362 | 2,747 | 56.94 | 54.99 |
| FL10500 | 13.67 | 195,862 | 68,786 | 296,764,305 | 473,676 | 2,075 | 54.90 | 63.22 |
| PA10500 | 14.86 | 252,918 | 93,497 | 419,364,592 | 533,373 | 2,658 | 56.48 | 59.05 |

­­

Table S2. Environmental parameters of water samples from the Mariana Trench

| Sampling Depth (m) | Temperature (°C) | Salinity | DO  (μmol/L) | pH | NO_x_^-^ (μmol/L) | NO_2_^-^ (μmol/L) | NH_4_ (nmol/L) | SiO_4_^2-^ (μmol/L) | PO_4_^3-^ (μmol/L) |
| --- | --- | --- | --- | --- | --- | --- | --- | --- | --- |
| 4^a^ | 29.82 | 34.06 | 193.64 | 8.24 | 0.10 | 0.02 | 18.90 | 0.55 | 0.02 |
| 50^a^ | 29.82 | 34.06 | 193.60 | 8.24 | 0.02 | 0.02 | 24.90 | 0.42 | 0.02 |
| 100^a^ | 28.62 | 34.05 | 197.50 | 8.26 | 0.03 | 0.02 | 26.70 | 0.56 | 0.10 |
| 125^a^ | 26.00 | 34.80 | 198.60 | 8.24 | 0.32 | 0.07 | 24.60 | 2.34 | 0.36 |
| 150^a^ | 12.95 | 34.47 | 131.30 | 7.93 | 5.12 | 0.11 | 20.60 | 5.05 | 1.12 |
| 200^a^ | 18.85 | 34.83 | 164.80 | 8.11 | 18.06 | 0.03 | 18.20 | 2.34 | 1.13 |
| 500^a^ | 11.47 | 34.70 | 89.67 | 7.74 | 32.76 | 0.02 | 21.00 | 19.66 | 2.19 |
| 800^a^ | 5.77 | 34.59 | 87.30 | 7.70 | 35.89 | 0.008 | 18.20 | 72.32 | 2.43 |
| 1000^a^ | 4.91 | 34.57 | 84.92 | 7.70 | 37.86 | 0.002 | 20.70 | 89.29 | 2.62 |
| 1500^a^ | 3.24 | 34.62 | 100.40 | 7.77 | 39.36 | 0.006 | 17.60 | 123.18 | 2.70 |
| 2000^a^ | 2.31 | 34.67 | 117.10 | 7.82 | 38.78 | 0.01 | 24.60 | 140.52 | 2.62 |
| 3000^a^ | 1.61 | 34.68 | 136.40 | 7.80 | 36.60 | 0.005 | 19.20 | 149.61 | 2.41 |
| 4000^a^ | 1.46 | 34.70 | 157.70 | 7.80 | 35.18 | 0.01 | 20.40 | 144.08 | 2.30 |
| 6050^a^ | 1.60 | 34.71 | 174.40 | 7.83 | 34.17 | 0.02 | 17.50 | 136.31 | 2.25 |
| 8320^a^ | 1.96 | 34.72 | 174.80 | 7.75 | 34.02 | 0.02 | 18.90 | 134.31 | 2.28 |
| 10257^b^ | ~1.00 | ~34.70 | 156.00 | ~7.80 | 36.43 | 0.03 | NA | NA | ~2.40 |

Note: a, from Mar. 2017 cruise; b, from Nunoura *et al*. [1]

Table S3. Relative abundance of the top 10 classes in metagenomic data based on NCBI-nr annotations across all samples

|  | FL0 | PA0 | FL4000 | PA4000 | FL9600 | FL10400 | PA10400 | FL10500 | PA10500 |
| --- | --- | --- | --- | --- | --- | --- | --- | --- | --- |
| Gammaproteobacteria | 19.11001 | 23.55075 | 31.34765 | 31.15879 | 38.40685 | 48.05872 | 50.2739 | 50.07003 | 48.24603 |
| Unclassfied | 34.54378 | 34.8875 | 38.60047 | 37.92544 | 31.41301 | 39.65018 | 40.68865 | 36.64315 | 37.18925 |
| Alphaproteobacteria | 27.35756 | 18.74716 | 19.44175 | 9.369488 | 23.52534 | 10.05372 | 5.653236 | 9.02756 | 9.336996 |
| Cyanobacteria | 13.90732 | 7.294868 | 0.613188 | 1.105375 | 0.380009 | 0.154881 | 0.123424 | 0.243006 | 0.27883 |
| Actinobacteria | 1.902579 | 0.964811 | 3.556752 | 6.204994 | 1.141612 | 0.470035 | 1.559009 | 0.697754 | 2.056671 |
| Bacilli | 0.175459 | 2.089166 | 1.162311 | 3.434832 | 0.132887 | 0.047392 | 0.055169 | 0.093559 | 0.101537 |
| Flavobacteriia | 0.756273 | 0.884414 | 0.3621 | 1.17062 | 2.668418 | 0.263135 | 0.424433 | 0.144643 | 0.409415 |
| Betaproteobacteria | 0.428998 | 1.728974 | 0.826612 | 0.63011 | 0.44388 | 0.34181 | 0.37589 | 1.688343 | 0.423996 |
| Deltaproteobacteria | 0.178784 | 0.305182 | 1.053333 | 0.906706 | 0.473361 | 0.554255 | 0.200628 | 0.560459 | 0.946632 |
| Planctomycetia | 0.372641 | 1.362779 | 0.207722 | 1.836255 | 0.080484 | 0.022609 | 0.020337 | 0.078367 | 0.063713 |

Table S4. Details of all the high-quality Metagenome assembled genomes (MAGs) constructed from contigs co-assembled from the four deepest water column samples. MAGs affiliated to *Oceanospirillales* are shown in bold type. The relative abundance of each MAG was normalized by that of *recA*.

| MAG ID | Marker lineage (Checkm) | Relative abundance in NBW (%) | Completeness  (%) | Contamination  (%) | Strain heterogeneity | Genome size (bp) | predicted genes | 16S rRNA gene length (bp) | 16S rRNA gene similarity (%) | EZBioCloud top-hit taxonomy |
| --- | --- | --- | --- | --- | --- | --- | --- | --- | --- | --- |
| **01** | c_Gammaproteobacteria (UID4444) | 11.57 ± 2.37 | 90.82 | 2.49 | 53.85 | 3,406,751 | 3088 | 1533 | 100% | Bacteria;Proteobacteria;Gammaproteobacteria;Oceanospirillales;Oceanospirillaceae;Oleibacter; Oleibacter marinus |
| 05 | k_Bacteria (UID3187) | 0.19 ± 0.11 | 84.97 | 2.68 | 0 | 3,591,271 | 3547 | 388 | 96.91 | Bacteria; Proteobacteria; Deltaproteobacteria; Bdellovibrionales; Bacteriovoracaceae; JF727692_g |
| **5.60** | c_Gammaproteobacteria (UID4443) | 0.46 ± 0.11 | 83.21 | 7.31 | 33.33 | 2,897,829 | 3144 | - | - | - |
| **12** | c_Gammaproteobacteria (UID4443) | 5.28 ± 7.71 | 93.21 | 2.02 | 0 | 4,720,602 | 4243 | - | - | - |
| 14 | c_Alphaproteobacteria (UID3422) | 0.41 ± 0.17 | 97.08 | 0.92 | 0 | 3,410,620 | 3398 | 92 | 100 | Bacteria; Proteobacteria; Alphaproteobacteria; Rhodobacterales; Hyphomonadaceae; Hyphomonas |
| 16 | c_Deltaproteobacteria (UID3216) | 0.12 ± 0.15 | 91.45 | 4.59 | 7.69 | 8,091,972 | 6970 | - | - | - |
| 18 | f_Rhodobacteraceae (UID3361) | 1.07 ± 0.57 | 97.82 | 1.34 | 14.29 | 5,053,848 | 4901 | - | - | - |
| 20 | k_Bacteria (UID3187) | 0.16 ± 0.09 | 93.45 | 8.48 | 0 | 4,162,313 | 4080 | 385 | 94.62 | Bacteria; Proteobacteria; Deltaproteobacteria; Bdellovibrionales; Bacteriovoracaceae; JF727692_g |
| 23 | k_Bacteria (UID2495) | 0.20 ± 0.28 | 98.9 | 0 | 0 | 2,098,732 | 2077 | 1477 | 90.47 | Bacteria; Proteobacteria; Alphaproteobacteria; Rhodospirillales; EU131034_f; EU131034_g |
| 26 | c_Gammaproteobacteria (UID4202) | 0.71 ± 0.67 | 97.71 | 0.91 | 0 | 3,348,910 | 2971 | 72 | 100 | Bacteria; Proteobacteria; Gammaproteobacteria; Enterobacteriales; Enterobacteriaceae; Brenneria |
| 27 | o_Actinomycetales (UID1593) | 0.16 ± 0.10 | 93.91 | 9.79 | 12.82 | 3,453,107 | 3506 | - | - | - |
| 32 | f_Rhodobacteraceae (UID3340) | 0.35 ± 0.27 | 98.86 | 0.45 | 0 | 5,220,098 | 4906 | - | - | - |
| 38 | k_Bacteria (UID2565) | 0.14 ± 0.13 | 94.32 | 1.7 | 0 | 3,594,013 | 2959 | 513 | 95 | Bacteria; Planctomycetes; Phycisphaerae; Phycisphaerales; Phycisphaeraceae; JF272039_g |
| 39 | s_algicola (UID2846) | 0.32 ± 0.20 | 99.14 | 0 | 0 | 2,951,163 | 2691 | - | - | - |
| 40 | c_Gammaproteobacteria (UID4444) | 0.08 ± 0.08 | 83.26 | 1.86 | 28.57 | 3,218,218 | 3219 | 581 | 96 | Bacteria; Proteobacteria; Gammaproteobacteria; Cellvibrionales; Spongiibacteraceae; Spongiibacter |
| 48 | o_Rhodospirillales (UID3754) | 0.11 ± 0.08 | 97.01 | 0.34 | 0 | 4,138,450 | 4043 | 369 | 100 | Bacteria; Proteobacteria; Alphaproteobacteria; Rhodospirillales; Rhodospirillaceae; Thalassospira |
| 52 | c_Gammaproteobacteria (UID4202) | 1.29 ± 0.61 | 98.79 | 0.54 | 0 | 3,678,075 | 3314 | 73 | 100 | Bacteria; Proteobacteria; Gammaproteobacteria; Enterobacteriales; Enterobacteriaceae; Brenneria |
| 59 | c_Alphaproteobacteria (UID3337) | 0.13 ± 0.05 | 98.3 | 3.6 | 0 | 4,654,292 | 4368 | 295 | 94.68 | Bacteria; Proteobacteria; Alphaproteobacteria; Parvularculales; Parvularculaceae; EU851414_g |
| 60 | c_Alphaproteobacteria (UID3422) | 1.35 ± 0.79 | 99.35 | 0 | 0 | 3,578,552 | 3403 | 101 | 100 | Bacteria; Proteobacteria; Alphaproteobacteria; Rhodobacterales; Hyphomonadaceae; Hyphomonas |
| 61 | k_Bacteria (UID203) | 0.11 ± 0.22 | 82.76 | 0 | 0 | 9,345,217 | 8824 | - | - | - |
| 63 | k_Bacteria (UID1453) | 0.12 ± 0.05 | 98.29 | 3.07 | 16.67 | 4,546,538 | 4235 | 1208 | 99.83 | Bacteria; Actinobacteria; Acidimicrobiia; Acidimicrobiales; Ilumatobacter_f; Ilumatobacter |
| 68 | o_Sphingomonadales (UID3310) | 0.08 ± 0.15 | 92.59 | 1.04 | 33.33 | 2,972,142 | 3063 | 340 | 100 | Bacteria; Proteobacteria; Alphaproteobacteria; Sphingomonadales; Sphingomonadaceae; Sphingopyxis |
| 69 | k_Bacteria (UID3187) | 0.18 ± 0.35 | 87.96 | 2.63 | 0 | 5,088,034 | 4750 | 370 | 91.29 | Bacteria; Proteobacteria; Deltaproteobacteria; OM27; U70713_f; U70713_g |
| 71 | c_Alphaproteobacteria (UID3422) | 0.62 ± 1.06 | 99.03 | 1.08 | 0 | 3,529,632 | 3450 | - | - | - |
| 72 | c_Deltaproteobacteria (UID3216) | 0.20 ± 0.22 | 80.99 | 3.71 | 0 | 6,768,871 | 5752 | 181 | 99.35 | Bacteria; Proteobacteria; Deltaproteobacteria; Bradymonadales; Bradymonadaceae; HM057789_g |
| **78** | c_Gammaproteobacteria (UID4444) | 0.70 ± 0.34 | 87.66 | 6.49 | 34.62 | 3,282,820 | 3189 | - | - | - |
| 94 | c_Alphaproteobacteria (UID3422) | 0.12 ± 0.04 | 98.38 | 1.87 | 33.33 | 3,215,486 | 3206 | 92 | 100 | Bacteria; Proteobacteria; Alphaproteobacteria; Rhodobacterales; Hyphomonadaceae; Henriciella |
| 97 | k_Bacteria (UID2495) | 0.25 ± 0.11 | 100 | 0 | 0 | 2,205,257 | 2118 | 311 | 90.07 | Bacteria; Proteobacteria; Alphaproteobacteria; Rhizobiales; Stappia_f; Labrenzia |
| 99 | c_Alphaproteobacteria (UID3422) | 0.11 ± 0.21 | 90.75 | 2.27 | 20 | 2,533,991 | 2651 | 101 | 100 | Bacteria; Proteobacteria; Alphaproteobacteria; Rhodobacterales; Hyphomonadaceae; Glycocaulis |
| 101 | c_Alphaproteobacteria (UID3337) | 0.22 ± 0.16 | 96.17 | 0.4 | 0 | 3,395,348 | 3321 | 294 | 97.73 | Bacteria; Proteobacteria; Alphaproteobacteria; Parvularculales; Parvularculaceae; EU851414_g |
| 103 | k_Bacteria (UID2565) | 0.12 ± 0.14 | 87.5 | 3.41 | 0 | 6,618,151 | 5266 | 1564 | 83.75 | Bacteria; FN550079_p; FN550079_c; FN550079_o; FN550079_f; FN550079_g |
| 106 | p_Bacteroidetes (UID2591) | 0.08 ± 0.10 | 92.17 | 4.5 | 8.33 | 8,810,728 | 6994 | 965 | 96.13 | Bacteria; Bacteroidetes; Sphingobacteriia; Sphingobacteriales; Saprospiraceae; Lewinella |
| 110 | k_Bacteria (UID3187) | 0.62 ± 0.65 | 91 | 1.79 | 0 | 3,514,822 | 3525 | - | - | - |
| 117 | k_Bacteria (UID2495) | 0.10 ± 0.09 | 87.61 | 1.1 | 100 | 1,586,662 | 1745 | 919 | 89.73 | Bacteria; Proteobacteria; Alphaproteobacteria; Sphingomonadales; Erythrobacteraceae; Altererythrobacter |
| 121 | c_Gammaproteobacteria (UID4445) | 0.16 ± 0.28 | 99.59 | 0.24 | 50 | 4,351,906 | 4064 | - | - | - |
| 122 | o_Rhizobiales (UID3449) | 0.17 ± 0.10 | 98.02 | 1.85 | 26.67 | 4,613,347 | 4448 | 170 | 97.06 | Bacteria; Proteobacteria; Alphaproteobacteria; Rhizobiales; Devosia_f; Cucumibacter |
| 123 | k_Bacteria (UID3187) | 0.28 ± 0.51 | 89.43 | 0.89 | 0 | 3,594,595 | 3484 | - | - | - |
| 124 | c_Alphaproteobacteria (UID3305) | 0.10 ± 0.20 | 90.09 | 1.09 | 33.33 | 1,647,322 | 1660 | - | - | - |
| 125 | c_Alphaproteobacteria (UID3337) | 0.10 ± 0.04 | 93.17 | 0 | 0 | 3,845,269 | 3634 | - | - | - |
| 126 | f_Rhodobacteraceae (UID3361) | 0.13 ± 0.05 | 91.52 | 1.66 | 0 | 3,754,555 | 3651 | - | - | - |
| 127 | o_Actinomycetales (UID1593) | 0.14 ± 0.08 | 90.48 | 1.01 | 0 | 2,215,730 | 2222 | - | - | - |
| 134 | c_Alphaproteobacteria (UID3305) | 0.14 ± 0.16 | 91.30 | 0.65 | 0 | 1,841,983 | 1944 | 1486 | 96.51 | Bacteria; Proteobacteria; Alphaproteobacteria; Rhodospirillales; Micavibrio_f; DQ395494_g |
| **156** | c_Gammaproteobacteria (UID4444) | 2.06 ± 3.77 | 97.84 | 3.57 | 0 | 4,429,143 | 4211 | - | - | - |

Table S5. The quantity of the bacterial population with *alkB* and *almA* at different depths. For each gene, values shown in the table are calculated as follows: Values in Supplementary Fig. 9b were multiplied by the relative abundance of bacterial genera containing each gene. The top five genera across all samples are shown.

| **Gene name** | **Genera** | **FL0** | **PA0** | **FL4000** | **PA4000** | **FL9600** | **FL10400** | **PA10400** | **FL10500** | **PA10500** |
| --- | --- | --- | --- | --- | --- | --- | --- | --- | --- | --- |
| *alkB* | Oleibacter | 3.06E+05 | 1.21E+05 | 8.99E+04 | 1.38E+05 | 9.79E+05 | 1.42E+07 | 1.69E+06 | 1.64E+07 | 2.23E+06 |
|  | Alcanivorax | 9.65E+05 | 5.04E+05 | 5.45E+05 | 1.99E+05 | 6.45E+05 | 2.07E+06 | 1.99E+05 | 1.25E+07 | 2.18E+05 |
|  | Pseudophaeobacter | 2.34E+06 | 1.87E+06 | 1.38E+05 | 3.23E+04 | 4.64E+05 | 4.53E+05 | 1.00E+05 | 5.03E+05 | 8.66E+04 |
|  | Oceanicaulis | 2.69E+06 | 1.62E+06 | 1.82E+05 | 3.70E+04 | 4.16E+05 | 5.94E+05 | 9.11E+04 | 4.63E+05 | 8.09E+04 |
|  | Marinobacter | 1.41E+05 | 1.07E+05 | 3.92E+05 | 1.84E+05 | 1.00E+06 | 1.50E+05 | 6.49E+04 | 6.79E+05 | 4.74E+05 |
| *almA* | Oleibacter | 5.99E+05 | 3.00E+05 | 2.53E+05 | 3.20E+05 | 2.15E+06 | 3.44E+07 | 3.80E+06 | 3.66E+07 | 4.98E+06 |
|  | Alcanivorax | 8.35E+05 | 4.81E+05 | 7.97E+05 | 2.72E+05 | 8.12E+05 | 4.45E+06 | 4.69E+05 | 2.31E+07 | 4.59E+05 |
|  | Alteromonas | 1.38E+06 | 6.73E+05 | 7.32E+05 | 2.41E+05 | 3.05E+06 | 1.59E+06 | 4.32E+04 | 2.36E+06 | 6.89E+05 |
|  | Pseudomonas | 4.31E+05 | 1.38E+05 | 4.96E+04 | 1.04E+05 | 3.66E+05 | 4.03E+06 | 3.66E+05 | 9.88E+05 | 2.84E+05 |
|  | Hydrocarboniphaga | 2.55E+05 | 2.77E+05 | 4.94E+05 | 3.39E+04 | 1.93E+06 | 1.69E+06 | 1.68E+05 | 1.03E+06 | 2.49E+05 |

Table S6. Details of all strains isolated from Challenger Deep 10,400 m water sample using an alkane-dependent medium. Strains tested for *n*-alkane degradation rates are shown in bold type.

| **Strain no.** | **Strains** | **Top-hit taxon** | **Similarity (%)** | **Taxonomy** |
| --- | --- | --- | --- | --- |
| ZYF842, ZYF843, **ZYF844**, ZYF846, ZYF847, ZYF849, ZYF850, ZYF853, ZYF856, ZYF857, ZYF858, ZYF862, ZYF464 | 14 | Alcanivorax jadensis | 99.14 | Bacteria; Proteobacteria; Gammaproteobacteria; Oceanospirillales; Alcanivoracaceae; Alcanivorax; Alcanivorax jadensis; |
| ZYF845, **ZYF848**, ZYF851, ZYF623 | 4 | Alcanivorax venustensis | 100 | Bacteria;Proteobacteria;Gammaproteobacteria;Oceanospirillales;Alcanivoracaceae;Alcanivorax; Alcanivorax venustensis |
| **ZYF854** | 1 | Alcanivorax dieselolei | 100 | Bacteria;Proteobacteria;Gammaproteobacteria;Oceanospirillales;Alcanivoracaceae;Alcanivorax; Alcanivorax dieselolei |
| ZYF671 | 1 | Halomonas janggokensis | 100 | Bacteria; Proteobacteria; Gammaproteobacteria; Oceanospirillales; Halomonadaceae; Halomonas; Halomonas janggokensis; |
| ZYF743 | 1 | Halomonas titanicae | 99.86 | Bacteria; Proteobacteria; Gammaproteobacteria; Oceanospirillales; Halomonadaceae; Halomonas; Halomonas titanicae; |
| ZYF855 | 1 | Idiomarina loihiensis | 100 | Bacteria;Proteobacteria;Gammaproteobacteria;Alteromonadales;Idiomarinaceae;Idiomarina; Idiomarina loihiensis |
| ZYF673, ZYF864 | 2 | Marinobacter salarius | 99.86 | Bacteria; Proteobacteria; Gammaproteobacteria; Alteromonadales; Marinobacter_f; Marinobacter; Marinobacter salarius; |
| ZYF859, ZYF863 | 2 | Marinobacter algicola | 100 | Bacteria;Proteobacteria;Gammaproteobacteria;Alteromonadales;Marinobacter_f;Marinobacter; Marinobacter algicola |
| ZYF669, ZYF860, ZYF861, ZYF678, ZYF679 | 5 | Marinobacter hydrocarbonoclasticus | 99.43 | Bacteria; Proteobacteria; Gammaproteobacteria; Alteromonadales; Marinobacter_f; Marinobacter; Marinobacter hydrocarbonoclasticus |
| ZYF672 | 1 | Neptunomonas phycophila | 99.86 | Bacteria; Proteobacteria; Gammaproteobacteria; Oceanospirillales; Oceanospirillaceae; Neptunomonas; Neptunomonas phycophila; |
| ZYF852 | 1 | Pseudohoeflea suaedae | 98.90 | Bacteria;Proteobacteria;Alphaproteobacteria;Rhizobiales;Phyllobacteriaceae;Pseudohoeflea; Pseudohoeflea suaedae |
| ZYF674, ZYF676, ZYF677, ZYF744, ZYF745 | 5 | Pseudomonas stutzeri | 99.71 | Bacteria; Proteobacteria; Gammaproteobacteria; Pseudomonadales; Pseudomonadaceae; Pseudomonas; Pseudomonas stutzeri; |

Table S7. *n*-Alkanes concentration (μg/gdw) in sinking particle and surface sediment samples.

| Samples | Depth (m) | C_14_ | C_15_ | C_16_ | C_17_ | C_18_ | C_19_ | C_20_ | C_21_ | C_22_ | C_23_ | C_24_ | C_25_ | C_26_ | C_27_ | C_28_ | C_29_ | C_30_ | C_31_ | C_32_ | C_33_ |
| --- | --- | --- | --- | --- | --- | --- | --- | --- | --- | --- | --- | --- | --- | --- | --- | --- | --- | --- | --- | --- | --- |
| Sinking particle | 2,000 | 0 | 0.52 | 0.60 | 0.77 | 0.58 | 0.51 | 0.46 | 0.40 | 0.413 | 0.38 | 0.37 | 0.37 | 0.40 | 0.41 | 0.25 | 0.37 | 0.20 | 0.12 | 0.12 | 0 |
|  | 4,000 | 0 | 3.00 | 4.24 | 3.58 | 2.91 | 2.70 | 3.63 | 3.99 | 4.42 | 3.74 | 1.93 | 1.43 | 0.59 | 0.92 | 0.27 | 0.73 | 0.24 | 0.41 | 0.31 | 0 |
|  | 6,000 | 0 | 1.23 | 1.81 | 2.70 | 2.83 | 3.31 | 3.37 | 3.09 | 2.29 | 1.31 | 0.65 | 0.29 | 0.23 | 0.29 | 0.12 | 0.34 | 0.17 | 0.14 | 0.05 | 0 |
| Surface sediment | 10,908 | 0 | 0 | 0.06 | 0.06 | 0.34 | 0.09 | 0.21 | 0.12 | 0.08 | 0.09 | 0.03 | 0.08 | 0.02 | 0.04 | 0.02 | 0.02 | 0.01 | 0.05 | 0.01 | 0.02 |
|  | 10,909 | 0 | 0 | 0.09 | 0.07 | 0.50 | 0.11 | 0.35 | 0.20 | 0.12 | 0.15 | 0.05 | 0.12 | 0.03 | 0.04 | 0.04 | 0.04 | 0.02 | 0.04 | 0.01 | 0.02 |
|  | 10,910 | 0.02 | 0.04 | 0.70 | 0.18 | 0.98 | 0.16 | 0.47 | 0.19 | 0.18 | 0.15 | 0.09 | 0.10 | 0.03 | 0.04 | 0.03 | 0.05 | 0.01 | 0.04 | 0.01 | 0.02 |

Table S8. Primer sequences used in this study.

| Targeted gene | Primer name | Primer sequence (5’-3’) | Function and reference |
| --- | --- | --- | --- |
| 16S rRNA gene | 515F | GTGYCAGCMGCCGCGGTAA | 16S rRNA gene high-throughput sequencing [5] |
|  | 806R | GGACTACNVGGGTWTCTAAT |  |
|  | B8F | AGAGTTTGATCCTGGCTCAG | Clone library construction [6] |
|  | B1510R | GGTTACCTTGTTACGACTT |  |
|  | Eub338F | ACTCCTACGGGAGGCAGCAG | qPCR [7] |
|  | Eub518R | ATTACCGCGGCTGCTGG |  |
| *alkB* | alkB-F | CGTCAACTACATCGAGCAYTAYGG | RT-qPCR |
|  | alkB-R | GCCGCTTAGGGTTNGCRTGRTG |  |
| *almA* | almA-F | TGACCGTCGAGGTGGAYGGNGARAC | RT-qPCR |
|  | almA-R | AGGTCCTCGGGCCARAAYTGNGG |  |

**References for supplementary information**

1. Nunoura T, Takaki Y, Hirai M, Shimamura S, Makabe A, Koide O, et al. Hadal biosphere, Insight into the microbial ecosystem in the deepest ocean on Earth. Proc Natl Acad Sci USA. 2015;112:E1230-E1236.

2. Tarn J, Peoples LM, Hardy K, Cameron J, Bartlett DH. Identification of free-living and particle-associated microbial communities present in hadal regions of the Mariana Trench. Front Microbiol. 2015;7:665.

3. Sun DL, Jiang X, Wu QL, Zhou NY. Intragenomic heterogeneity in 16S rRNA genes causes overestimation of prokaryotic diversity. Appl Environ Microb. 2013;79:5962-5969.

4. Wang WP, Shao ZZ. The long-chain alkane metabolism network of *Alcanivorax dieselolei*. Nat Commun. 2014;5:5755.

5. Walters W, Hyde ER, Berg-Lyons D Ackermann G, Humphrey G, Parada A, et al. Improved bacterial 16S rRNA gene (V4 and V4-5) and fungal internal transcribed spacer marker gene primers for microbial community surveys. Msystems. 2016;1:e00009-15.

6. Weisburg WG, Barns SM, Pelletier DA, Lane DJ. 16S ribosomal DNA amplification for phylogenetic study. J Bacteriol. 1991;173:697-703.

7. Yin Q, Fu B, Li B, Shi X, Inagaki F, Zhang XH. Spatial variations in microbial community composition in surface seawater from the ultra-oligotrophic center to rim of the South Pacific Gyre. PLoS One. 2013;8:e55148.
