# Supplementary material for: Proliferation of hydrocarbon-degrading microbes at the bottom of the Mariana Trench
Source: Microbiome. 2019 Apr 12;7:47. doi: 10.1186/s40168-019-0652-3 (PMC6460516; doi:10.1186/s40168-019-0652-3)
Supplement: Supplementary file 2 — Supplementary methods (DOCX 33 kb) [file 40168_2019_652_MOESM2_ESM.docx]

**Additional file 2**

**Proliferation of hydrocarbon degrading microbes at the bottom of the Mariana Trench**

Jiwen Liu^1,2†^, Yanfen Zheng^1†^, Heyu Lin^1^, Xuchen Wang^3^, Meng Li^4^, Yang Liu^4^, Meng Yu^3^, Meixun Zhao^2,3^, Nikolai Pedentchouk^5^, David J. Lea-Smith^6^, Jonathan D. Todd^6^, Clayton R. Magill^7^, Wei-Jia Zhang^8^, Shun Zhou^1^, Delei Song^1^, Haohui Zhong^1^, Yu Xin^2,3^, Min Yu^1,2^, Jiwei Tian^9,10*^, Xiao-Hua Zhang^1,2*^

Corresponding author: Xiao-Hua Zhang

Email: [xhzhang@ouc.edu.cn](mailto:xhzhang@ouc.edu.cn)

**Supplementary Methods**

**Sinking particle sampling and environmental measurement**

Seawater nutrients were analyzed using filtrates through 0.4 μm polycarbonate members in a AA3 autoanalyser system. Dissolved oxygen (DO) was measured immediately upon retrieval by the Winkler method. The final DO concentration was calibrated by pressure and temperature. pH was measured by a Mettler-Toledo pH meter onboard and was corrected for temperature. For sinking particles collection, each sample cup collected one month of sinking particles for a total of 27 separate samples (3 depths, 9 months). Samples cups containing particles and seawater were treated with mercuric chloride (3%, w/v). Upon recovery, trap samples were stored at 5 °C until analysis.

**Experimental test of bacterial *n*-alkane degradation**

Three *Alcanivorax* strains (*A*. *jadensis* ZYF844, *A. venustensis* ZYF848 and *A. dieselolei* ZYF854) were cultured in 50 ml MMC medium in 250-ml erlenmeyer flasks at 4 °C on a rotary shaker (170 rpm) for 30 days. 10 μl of alkane mixture (C_8_, C_9_, C_10_, C_11_, C_12_, C_13_, C_14_, C_15_, C_16_, C_17_, C_18_, C_19_, C_20_, C_22_, C_24_, C_26_, C_28_, C_30_, C_32_ and C_36,_ 500 μg/ml of each component, purchased from Supelco) were added to 50 ml of MMC medium. Medium without bacteria was used as a negative control. In order to test whether they could degrade *n*-alkanes at high pressure, species were cultured at 4 °C, 60 MPa for 20 days. High pressure incubations were conducted in stainless steel reactors (Nantong Feiyu Oil Science and Technology Exploitation, China). Pressure was delivered by water using a manual pump. Alkanes (<C_18_) could not be detected at the end of this period, likely due to volatilization during this incubation period. A 5 mg aliquot of *n*-eicosane was added to 5 ml of MMC medium when appropriate. Medium containing an equal amount of *n*-eicosane without bacteria was used as a negative control. *Oleibacter marinus* DSM 24913 (=NBRC 105760) was bought from the Marine Culture Collection of China, and demonstrated poor growth at low temperature and high pressure. Thus, this strain was cultivated at 16 °C with *n*-eicosane as a sole carbon source.

***n*-Alkane profiling and concentration measurement**

The alkane extracts of two rounds of extraction was combined, evaporated to near dryness using a Buchi Professional Multivapor (P-12) and then re-dissolved in 5 ml of hexane. The *n*-alkanes were separated using a 1 × 20 cm glass chromatography column packed with activated silica gel (100-200 mesh) and eluted with 25 ml of hexane. The eluate was evaporated down to approximately 1 ml, then transferred to a glass vial and further concentrated down to 100 μl with high purity N_2_ for *n*-alkane analysis. *n*-Alkanes were analyzed using an Agilent 7890B Gas Chromatography (GC) with an FID detector. *n*-Alkanes were separated using a HP-1 column (50 m × 0.32 mm × 0.17 μm) programmed from 50 ^o^C to 300 ^o^C at a rate of 3 ^o^C/min and held at 300 ^o^C for 30 min. Helium was used as a carrier gas, and the flow rate was set at 1 ml/min. Individual *n*-alkanes were identified based on the retention times of *n*-alkanes standards (Sigma), and the concentrations of each *n*-alkane were calculated based on the standard calibration curve of each corresponding standard. The recovery efficiency for the internal standard was 92 ± 5%, therefore, the *n*-alkanes concentrations were not corrected for standard recovery.

**Details for characterization of carbon and hydrogen isotopic compositions of *n*-alkanes**

The chromatograph oven was programmed from 60 °C (1 min) to 320 °C (20 min) at 6 °C min^–1^ with helium as a carrier. Biomarker *n*-alkanes were injected in splitless mode onto a 30-m TG-5SilMS (0.25 mm × 0.25 µm) fused silica column prior to either combustion over nickel and platinum wire with 1% oxygen in helium at 1000 °C (GC-C-irMS) or pyrolysis over graphite at 1420 °C (GC-pyr-irMS). Isotopic values are shown relative to reference gas calibrated to either Vienna Pee Dee Belemnite (VPDB, δ^13^C) or Vienna Standard Mean Ocean Water (VSMOW, δ^2^H) in per mil (‰) units. Within-run accuracy and precision (1σ s.d.) were determined from co-injected bracket standards of known isotopic composition (Mix B4, C_16_-C_30_ *n*-alkanes; Schimmelmann Standards, Indiana University). Respective accuracy/precision was equal to 0.07/0.10‰ for δ^13^C and 4.4/9.0‰ for δ^2^H (*n* = 304).
